# A HIF1α Regulatory Loop Links Hypoxia and Mitochondrial Signals in Pheochromocytomas

**DOI:** 10.1371/journal.pgen.0010008

**Published:** 2005-07-25

**Authors:** Patricia L. M Dahia, Ken N Ross, Matthew E Wright, César Y Hayashida, Sandro Santagata, Marta Barontini, Andrew L Kung, Gabriela Sanso, James F Powers, Arthur S Tischler, Richard Hodin, Shannon Heitritter, Francis Moore, Robert Dluhy, Julie Ann Sosa, I. Tolgay Ocal, Diana E Benn, Deborah J Marsh, Bruce G Robinson, Katherine Schneider, Judy Garber, Seth M Arum, Márta Korbonits, Ashley Grossman, Pascal Pigny, Sérgio P. A Toledo, Vania Nosé, Cheng Li, Charles D Stiles

**Affiliations:** 1 Department of Cancer Biology, Dana-Farber Cancer Institute, Harvard Medical School, Boston, Massachusetts, United States of America; 2 Broad Institute, Massachusetts Institute of Technology, Cambridge, Massachusetts, United States of America; 3 University of São Paulo School of Medicine, São Paulo, Brazil; 4 Brigham and Women's Hospital, Boston, Massachusetts, United States of America; 5 Center of Endocrine Investigations, Hospital de Niños R. Gutierrez, Buenos Aires, Argentina; 6 Department of Pediatric Oncology, Dana-Farber Cancer Institute, Harvard Medical School, Boston, Massachusetts, United States of America; 7 Tufts–New England Medical Center, Boston, Massachusetts, United States of America; 8 Massachusetts General Hospital, Boston, Massachusetts, United States of America; 9 Yale University, New Haven, Connecticut, United States of America; 10 Royal North Shore Hospital and Kolling Institute of Medical Research, University of Sydney, Australia; 11 Division of Population Sciences, Dana-Farber Cancer Institute, Harvard Medical School, Boston, Massachusetts, United States of America; 12 Boston Medical Center, Boston, Massachusetts, United States of America; 13 St. Bartholomew's Hospital, London, United Kingdom; 14 Regional University Hospital, Lille, France; 15 Department of Biostatistical Science, Dana-Farber Cancer Institute, Harvard Medical School, Boston, Massachusetts, United States of America; The Jackson Laboratory, United States of America

## Abstract

Pheochromocytomas are neural crest–derived tumors that arise from inherited or sporadic mutations in at least six independent genes. The proteins encoded by these multiple genes regulate distinct functions*.* We show here a functional link between tumors with *VHL* mutations and those with disruption of the genes encoding for succinate dehydrogenase (SDH) subunits B (SDHB) and D (SDHD). A transcription profile of reduced oxidoreductase is detected in all three of these tumor types, together with an angiogenesis/hypoxia profile typical of VHL dysfunction. The oxidoreductase defect, not previously detected in *VHL*-null tumors, is explained by suppression of the SDHB protein, a component of mitochondrial complex II. The decrease in SDHB is also noted in tumors with *SDHD* mutations. Gain-of-function and loss-of-function analyses show that the link between hypoxia signals (via VHL) and mitochondrial signals (via SDH) is mediated by HIF1α. These findings explain the shared features of pheochromocytomas with *VHL* and *SDH* mutations and suggest an additional mechanism for increased HIF1α activity in tumors.

## Synopsis

Pheochromocytomas (also known as paragangliomas) are highly vascular tumors that arise from mutations in a diverse and apparently unrelated group of tumor suppressor genes and oncogenes. The authors show here that three of the genes that cause hereditary pheochromocytomas have a common function. Specifically, these genes, *VHL,* *SDHB,* and *SDHD,* encode proteins that regulate a transcription factor known as hypoxia-inducible factor 1 subunit α (HIF1α), which helps cells adapt to hypoxia (low oxygen levels). *VHL* is named after its role in von Hippel-Lindau disease (VHL), an inherited disorder that predisposes individuals to pheochromocytomas and other tumors. Previous studies showed that when cells lack *VHL,* HIF1α is not degraded, resulting in a signal that resembles hypoxia. The authors found that loss of two genes that cause two distinct pheochromocytoma syndromes (the genes *SDHB* and *SDHD*, which encode the subunits B and D of succinate dehydrogenase, a component enzyme of the energy and respiratory system in mitochondria) also triggers a HIF1α response. The researchers further discovered that high H1F1α levels can suppress SDHB. This suggests a regulatory loop that further enhances the “hypoxia” profile of tumors. This finding provides a rational explanation for the shared features of these distinct syndromes and may be relevant for other cancers with a prominent hypoxic pattern.

## Introduction

Adrenal and extra-adrenal pheochromocytomas (also known as paragangliomas) are catecholamine-secreting tumors derived from chromaffin cells of neural crest origin [1]. Pheochromocytomas can arise as a result of mutations in the following disease-associated genes: *RET* in multiple endocrine neoplasia type 2 (MEN2); *VHL* in von Hippel-Lindau disease (VHL); *NF1* in neurofibromatosis type 1 (NF1); and *succinate dehydrogenase (SDH)* *subunits B, C,* or *D* in familial paraganglioma syndromes type 4 (PGL4), type 3 (PGL3), and type 1 (PGL1), respectively [2]. The various pheochromocytoma susceptibility genes modulate a variety of signaling pathways that are superficially unrelated to one another. However, the uniform phenotype of the tumors that arise from these distinct genetic lesions suggests the presence of underlying biochemical links.

The *VHL* tumor suppressor is a key mediator of the hypoxia response. It targets the hypoxia-inducible factor 1 subunit α (HIF1α) for ubiquitin-mediated degradation under normal oxygen conditions [3]. HIF1α has been shown to be critical for the oncogenic effects resulting from *VHL* mutations in specific cellular contexts [4,5]. Two other genes related to familial paraganglioma, *SDHB* and *SDHD,* encode subunits of SDH, the enzyme that composes mitochondrial complex II [6,7]. This enzyme is both a component of the Krebs cycle, by oxidizing succinate to fumarate, and of the mitochondrial respiratory chain, by transferring electrons to the ubiquinone pool [8]. Familial paragangliomas associated with *SDHB* and *SDHD* mutations resemble the carotid body growths that occur as a result of chronic hypoxia exposure in individuals living at high altitudes [6]. These clinical observations and the finding of increased expression of HIF targets in tumors with SDH mutations [9,10] have suggested the possibility that the *VHL* and *SDH* syndromes intersect at the molecular level.

As an entry-level screen for interacting signals, we generated global expression signatures of 76 hereditary and sporadic primary pheochromocytomas and paragangliomas. We show here that pheochromocytomas with *VHL* and *SDHB* or *SDHD* mutations form a tight cluster with a clear hypoxia and reduced oxidoreductase signature. This observation led to the identification of suppressed SDHB protein in tumors with *VHL* mutation and to the genetic demonstration that this effect is HIF-dependent. Our findings link pheochromocytomas with mutations in distinct genes—*VHL,* *SDHB,* and *SDHD*—and suggest that mitochondrial complex II inhibition contributes to development of pheochromocytomas with *VHL* mutation.

## Results

### Expression Profiling Links Pheochromocytomas with *VHL* and *SDHB* or *SDHD* Mutations

**Figure 1:**
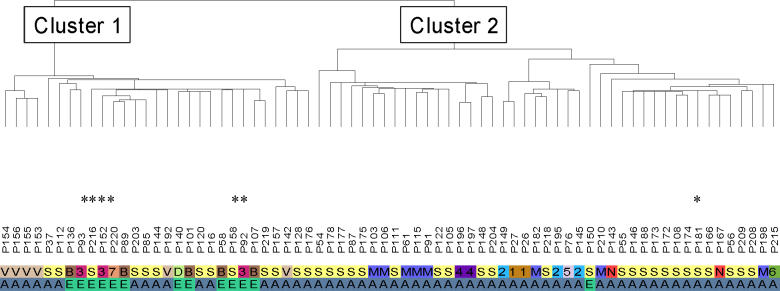
Unsupervised Analysis of Pheochromocytomas Links Tumors with *VHL* and *SDHB* or *SDHD* Mutations Unsupervised hierarchical clustering identifies two major clusters in pheochromocytomas: Cluster 1 contains VHL (V), SDHD (D), and SDHB (B) tumors; Cluster 2 contains MEN2 (M) and NF1 (N) pheochromocytomas. Multiple tumors from seven independent unclassified families with recurrent pheochromocytoma (numbered 1–7) and also sporadic tumors (S) are distributed between the two clusters. Letters or numbers on the first row indicate the various tumor classes, as described above. The second row identifies tumor location as adrenal (A) or extra-adrenal (E). Mutations were later detected in samples marked with an asterisk, guided by cluster distribution (see text and Table 1 for details).

Unsupervised hierarchical cluster analysis of a cohort of 76 sporadic and hereditary pheochromocytomas (Dataset S1) identified two dominant expression clusters (Figure 1; Dataset S2). Cluster 1 comprised all VHL and SDH tumors. Cluster 2 contained all MEN2 and NF1 pheochromocytomas. The remaining unknown familial tumors and 37 sporadic samples were partitioned into one or the other of the two major clusters. Cluster 1 contained 12 of the 13 extra-adrenal tumors. However, the bipartite distribution of tumor sets is not a simple reflection of anatomical location of the tumor because more than half of Cluster 1 tumors are adrenal in origin (Figure 1).

**Table 1:**
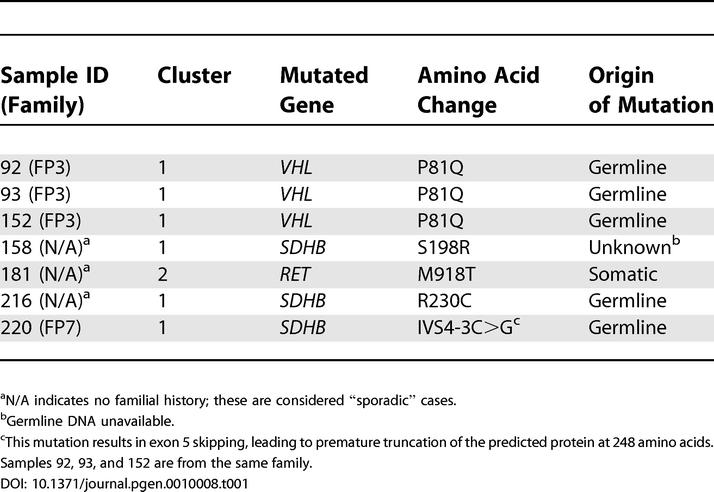
Mutations Identified in Previously Unclassified Hereditary and Sporadic Pheochromocytomas and Their Association with Expression Cluster Distribution

^a^N/A indicates no familial history; these are considered “sporadic” cases.

^b^Germline DNA unavailable.

^c^This mutation results in exon 5 skipping, leading to premature truncation of the predicted protein at 248 amino acids. Samples 92, 93, and 152 are from the same family.

To validate the expression clusters, we initially confirmed the expression difference between the two clusters by quantitative real-time PCR or Western blot analysis of genes identified by the unsupervised analysis (Dataset S3). Next, we sequenced all familial samples and also 20 of the sporadic tumors for mutations in known pheochromocytoma-associated genes. We detected novel *VHL,* *SDHB,* and *RET* mutations in six samples derived from four independent families (Table 1). In all cases, the mutations resided in component genes predicted by the cluster distribution.

**Figure 2:**
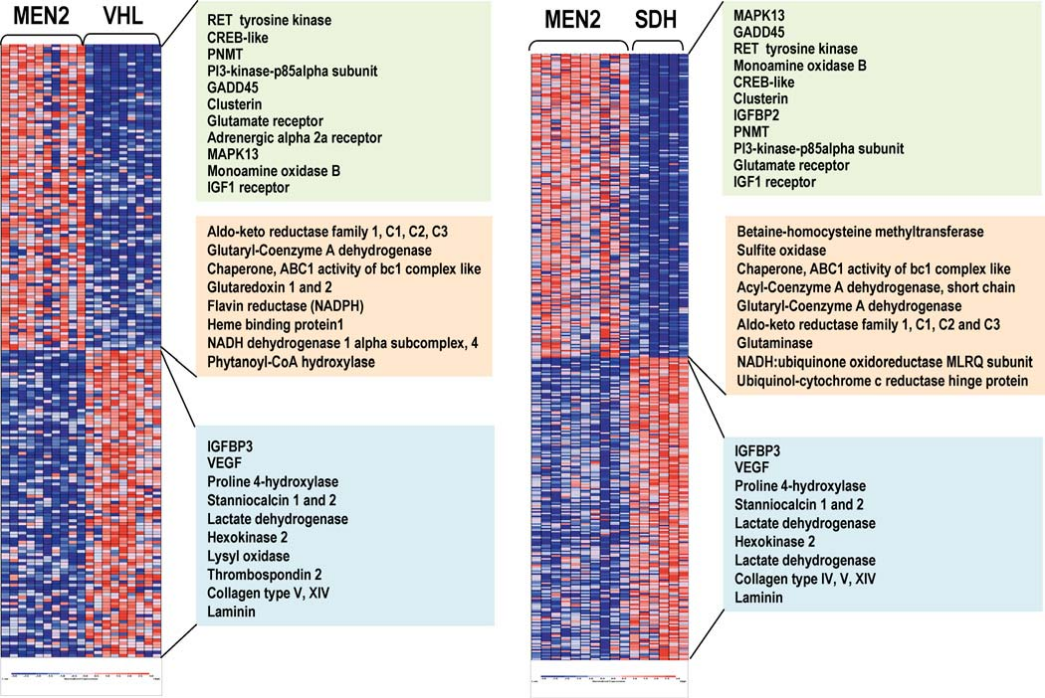
Similarity between VHL and SDH Tumors from Cluster 1 by Supervised Learning Methods Supervised analysis reveals an extensive overlap between genes that discriminate MEN2 from VHL (left) and MEN2 from SDH tumors (right). Samples are shown in columns and genes are represented in rows. Expression levels are normalized for each gene, where the mean is zero. Red indicates high-level expression and blue, low-level expression. The color scale at the bottom indicates relative expression and standard deviations from the mean. Some representative genes are displayed in a color-coded manner according to their functional classes (green, kinase receptor signaling and adrenergic metabolism; pink, oxidative response; blue, hypoxia-responsive/angiogenesis genes). Within each of these functional classes the order of gene appearance in the heat map has been maintained for each class comparison. Complete gene lists are available as Dataset S4.

All VHL tumors, including the newly identified mutated samples in Cluster 1, as well as the MEN2 tumors of Cluster 2, were used to generate two-class predictors (Dataset S4). Likewise, gene predictors were also created by comparing MEN2 and another component of Cluster 1, SDH tumors (including both *SDHB* and *SDHD* mutants). An extensive overlap was seen between the genes that discriminate MEN2 from SDH tumors and those that distinguish MEN2 from VHL tumors (Figure 2; Dataset S4). Over 92% of the entire sample cohort was correctly assigned to one of the two classes, in agreement with the unsupervised clustering distribution (Dataset S4). These results outline the molecular similarities between subcomponent tumors of Cluster 1 (VHL and SDH) and validate the two expression clusters detected by the unsupervised analysis (see Figure 1).

### Suppressed SDHB Expression Is a Common Feature of VHL and SDH Tumors

Cluster 1 tumors are associated with a set of biological programs that differs markedly from Cluster 2 pheochromocytomas (Table 2; Figure 2; Dataset S4). These tumors display a rich signature of angiogenesis, hypoxia, extracellular matrix elements, and coordinated suppression of oxidoreductase enzymes. The first three components have been associated with mutations in the *VHL* gene, whose product is a known regulator of the activity of the HIF pathway [11]. Of note, pheochromocytoma was the sole manifestation in one of the VHL families in this series (tumors 155 and 156; see Figure 1). These features are suggestive of VHL type 2C, which has been deemed HIF-independent [12,13]. These tumors clustered with the remaining VHL samples in the expression profiling analysis, suggesting that transcription similarities between these cases and the remaining VHL tumors outnumber differences that they may bear. However, only long-term follow-up, not available in this kindred, can precisely define these tumors as VHL type 2C. The similar transcription profile of pheochromocytomas with mutations in *SDH* subunits indicates that the mechanism by which these tumors develop also involves the hypoxia-sensing pathway. Owing to the limited amount of tumor material, we were not able to quantitate HIF1α and HIF2α protein levels in our primary samples.

**Table 2:**
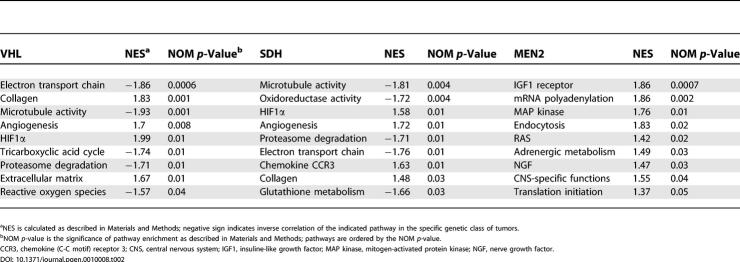
Gene-Set Enrichment Analysis (GSEA) of Pathways Significantly Represented in Three Genetic Classes of Pheochromocytoma

^a^NES is calculated as described in Materials and Methods; negative sign indicates inverse correlation of the indicated pathway in the specific genetic class of tumors.

^b^NOM *p-*value is the significance of pathway enrichment as described in Materials and Methods; pathways are ordered by the NOM *p-*value.

CCR3, chemokine (C-C motif) receptor 3; CNS, central nervous system; IGF1, insuline-like growth factor; MAP kinase, mitogen-activated protein kinase; NGF, nerve growth factor.

**Figure 3:**
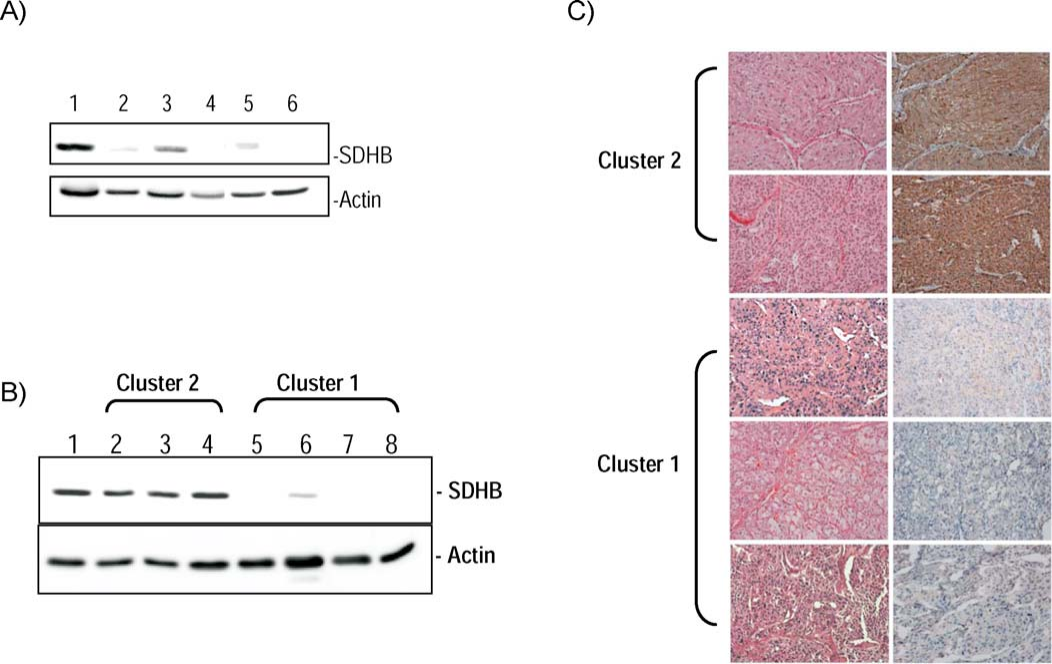
Low Expression of SDHB Is a General Feature of Cluster 1 Tumors (A) Expression of SDHB protein in pheochromocytomas with *SDHB* or *SDHD* mutations. Western blot analysis of SDHB of whole cell lysates from primary tumors was performed as described in Methods. Lane 1 is normal adrenal medulla used as control and lanes 2–6 are tumors 140, 158, 136, 58, and 220, respectively, from Figure 1 and Table 1. β-actin was used as a loading control. (B) SDHB expression segregates with cluster membership. Cluster 2 tumors, comprising MEN2, NF1, and other sporadic tumors, are shown in lanes 2–4 (tumors 105, 91, and 196, respectively, from Figure 1). Cluster 1 contains tumors with *VHL* and *SDHB* mutations and a subset of sporadic samples (lanes 5–7 are tumors 16, 85, 101, and 152, respectively, from Figure 1). Lane 1 is normal adrenal medulla. β-actin was used as a loading control. (C) Immunostaining of SDHB protein in pheochromocytomas or paragangliomas with various genetic backgrounds. A MEN2-related pheochromocytoma is shown on the top row, followed by tumors with mutations in *NF1, SDHB, SDHD,* and *VHL* genes. Corresponding hematoxylin/eosin staining is shown on the left.

Another element of the Cluster 1 signature—a synchronized suppression of mitochondrial functions—was noted by predominantly reduced expression of components of the oxidative response and Krebs cycle in both the unsupervised and supervised analyses of these tumors. This profile generated multiple significant scores by the pathway-enrichment analysis method, gene set enrichment analysis (GSEA) (Table 2), which measures the degree of inverse correlation of oxidative pathways of a rank-ordered gene list derived from the pairwise comparisons. Depressed oxidoreductase function has not been previously linked to a HIF-mediated signature. Mitochondrial complex II is a component of the electron transport chain, and mutations of *SDHB* or *SDHD* genes that abrogate the oxidoreductase function of complex II can cause pheochromocytomas [9,14,15]. Because of the role of SDH subunits as tumor suppressors, we reasoned that the oxidoreductase signature observed in pheochromocytomas from Cluster 1 (Table 2) might indicate that complex II disruption could contribute to other tumors besides those with *SDH* mutations. This prompted us to examine the link between pheochromocytomas with *VHL* and *SDH* mutations in our series by first determining the protein expression of the catalytic unit of complex II, SDHB. We found that the expression of SDHB is reduced in all tumors with *SDH* (both *SDHB* and *SDHD*) mutations in this cohort (Figure 3A), indicating that low SDHB expression functions as a surrogate for disruption of complex II. Importantly, suppressed SDHB levels were also found in the majority of tumors with *VHL* mutations and sporadic pheochromocytomas from Cluster 1 tested by immunoblots (Figure 3B). To confirm this finding, we performed immunostaining of SDHB in pheochromocytomas or paragangliomas representative of the various genetic syndromes using available paraffin-embedded material. SDHB immunostaining was highly concordant with the immunoblot findings (Figure 3C), suggesting that SDHB downregulation may be a feature of a broader group of pheochromocytomas. Although SDHB mRNA was collectively lower in Cluster 1 tumors than in Cluster 2 pheochromocytomas (Dataset S5), levels of the SDHB protein did not exactly parallel mRNA abundance in individual tumor samples, suggesting that a transcription defect cannot entirely account for the differences in SDHB expression observed at the protein level.

In contrast, Cluster 2 tumors exhibited a distinct set of biological programs, including genes that mediate translation initiation, protein synthesis, and kinase signaling (Table 2; Dataset S4). The two prototype genes of Cluster 2 *(RET* and *NF1)* are linked by their common ability to activate the RAS/RAF/MAP kinase signaling cascade [16,17]. Activated RAS signaling has been shown by expression profiling to be associated with increased translation events [18]. Thus, the anabolic functions of activated RAS may constitute the biochemical mechanism that underlies the assignment of MEN2 and NF1 tumors to Cluster 2. Increased expression of genes defining a neural/neuroendocrine profile and adrenergic metabolism were also prominent features of this cluster (Table 2; Dataset S4).

### HIF1α Contributes to SDHB Regulation

**Figure 4:**
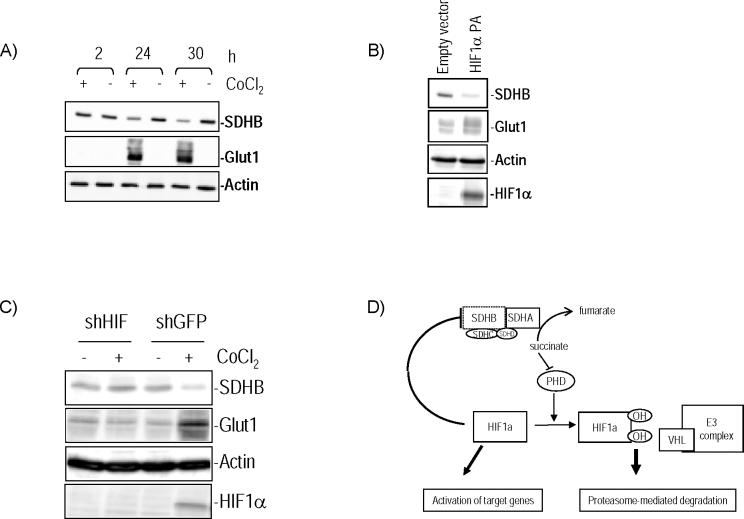
HIF1α Attenuates SDHB Levels (A) HIF1α expression was induced by treatment of mouse pheochromocytoma MPC 9/3L cells with 150 μM cobalt chloride for the indicated times. SDHB expression decreased in treated cells. Glut1 indicates increased activity of HIF1α, and β-actin was used as a loading control. (B) Transient expression in HEK293 cells of a HIF1α double mutant PA (P402A/P564A) that is resistant to VHL-mediated degradation reduced expression of SDHB. (C) A2058 cell lines stably expressing HIF1α shRNA do not show change in SDHB after cobalt chloride exposure, while SDHB is downregulated in control GFP shRNA cells treated with cobalt chloride. (D) Proposed model of HIF1α and SDHB interregulation. HIF1α downregulates SDHB, which leads to complex II dysfunction. High succinate levels resulting from loss of complex II, in turn, inhibit prolyl hydroxylase (PHD) activity [19]. Non-hydroxylated HIF1α is resistant to VHL-mediated targeting for degradation and can therefore activate downstream genes, such as angiogenic factors. “E3 complex” indicates the E3 ubiquitin ligase complex for which VHL is the substrate recognition factor.

Because of the critical role of VHL in controlling availability of HIF in normoxic conditions, we next investigated whether downregulation of SDHB was HIF-dependent. In two cell line models, HEK293 and mouse pheochromocytoma cell line (MPC) 9/3L, exposure to the hypoxia-mimetic agent cobalt chloride reduced SDHB protein expression (Figure 4A). Further, transient expression of a mutant, nondegradable form of HIF1α, HIF1αP402A/P564A, was able to downregulate SDHB (Figure 4B). In contrast, suppression of HIF1α in neural crest–derived A2058 melanoma cell lines stably expressing HIF1α short hairpin RNA (shRNA) prevented the reduction of SDHB after exposure to cobalt chloride (Figure 4C), in contrast to cells expressing a control shRNA sequence, supporting a central role of HIF1α in regulating SDHB levels. This finding implicates HIF1α as a mediator of the clustering between *VHL*- and *SDH*-mutated primary pheochromocytomas identified in our expression profiling studies. Similar to what we found in primary pheochromocytomas, no decrease in SDHB mRNA was detected when these cell lines were treated with hypoxia-mimetic drugs (data not shown), suggesting that a posttranscriptional phenomenon is related to these findings.

## Discussion

Transcription profiling of a large series of primary pheochromocytomas reveals that tumors with *VHL* and *SDH* mutations are closely linked. The hypoxia-angiogenesis signature identified by our analysis of primary tumors with *SDHB* or *SDHD* mutations confirms and extends recent observations on the role of SDH proteins in cultured cell lines. Selak et al. showed that disruption of the mitochondrial complex II results in increased HIF1α activity and that this upregulation is channeled through inhibition of prolyl hydroxylase function [19]. This hydroxylation step is essential for VHL-dependent HIF1α degradation [20,21]. Our data show that mitochondrial complex II mutations lead to upregulation of HIF1α targets in human tumor tissue and indicate an additional level of interplay between the SDHB and HIF1α proteins, i.e., a reciprocal effect of HIF1α in modulating components of the mitochondrial complex II. Our findings favor the existence of an autoregulatory loop whereby HIF1α contributes to attenuation of SDHB levels, resulting in complex II inhibition (Figure 4D). High levels of succinate resulting from loss of complex II function can in turn block HIF1α degradation through inhibition of prolyl hydroxylases. However, while succinate accumulation, but not oxidative stress, was considered the oncogenic trigger by Selak et al. [19], increased levels of reactive oxygen species have been reported in animal models of SDHC dysfunction [22–24]. The latter results are consistent with the oxidoreductase defect of our primary tumor samples. The precise mechanisms for the interaction between HIF1α and SDHB still remain to be identified, but our data suggest that a posttranscriptional response is likely to be involved. Of note, and in keeping with our current data, HIF2α /EPAS1-null mice were reported to have increased SDH activity in muscle [25].

One provocative possibility suggested by these findings is that the tumorigenic effects of VHL mutations in chromaffin tissue might involve dysfunction of mitochondrial complex II. Hence, we propose that in VHL-derived tumors two complementary mechanisms play a role in stabilizing HIF1α: the loss of VHL-dependent targeting of HIF1α for proteasome-mediated degradation, and a second mechanism that is dependent on low levels of HIF1α hydroxylation resulting from complex II dysfunction. The effects of HIF1α in our model were less marked than those observed with hypoxia-mimetic agents, which inhibit prolyl hydroxylases. This suggests that additional factors, besides HIF1α, might be involved in SDHB suppression. As such, it will be relevant to determine how SDHB and mitochondrial complex II are regulated in VHL type 2C variants that have been proposed to impart distinct, HIF-independent signaling outcomes [12,13].

A relationship between SDH function and oxygen regulation has been suspected based on previous identification of increased expression of angiogenic factors in cases of *SDH*-mutant pheochromocytomas [9]. Also, clinical similarities besides pheochromocytoma have been noted in families with germline mutations of *VHL* and *SDHB* [26]. This is in agreement with our transcription results and biochemical data indicating that HIF1α is involved in this association. The bipartite transcription clustering of pheochromocytomas has thus provided an explanation for the link between two genetic subtypes of pheochromocytomas. We also showed that this distribution has high predictive value, as determined by the identification of previously undetected mutations in tumor samples segregating with the appropriate cluster. The successful distinction of tumors from Cluster 1 and Cluster 2 by SDHB immunostaining in our pilot series suggests that this may be developed into a new screening method to classify pheochromocytomas in one of two major categories that reflect the underlying genetic defect. Of interest, in a recent study, immunohistochemistry of head and neck paragangliomas with *SDHB* and *SDHD* mutations revealed similar suppression of SDHB, which was accompanied by morphologically abnormal mitochondria [27]. This is in line with our results of catecholamine-secreting tumors and suggests that SDHB downregulation is a general marker of complex II dysfunction. This study also describes a number of sporadic head and neck paragangliomas with low SDHB staining; these tumors might correspond with Cluster 1 pheochromocytomas for which no detectable mutation was identified and that also appear to arise from disruption of related pathways. It remains to be tested whether the predominant hypoxic-angiogenic profile of pheochromocytomas with *VHL* and *SDH* mutations will render these tumors targets for antiangiogenic therapies. This will be particularly relevant for *SDHB*-mutant pheochromocytomas which have been suggested to be more prone to malignancy [10].

## Materials and Methods

### 

#### Tumor specimens.

Tumor samples were obtained from patients with catecholamine-secreting pheochromocytomas and thoracic or abdominal paragangliomas according to institutionally approved protocols. Fragments were obtained from the core of the tumor and contained more than 70% tumor cells. Samples with a clear adjacent cortical component were macrodissected. Specimens were snap-frozen at time of surgical resection and stored at −70 °C or in liquid nitrogen until processed.

Diagnosis of pheochromocytoma and/or paraganglioma was confirmed by histology in every case. Heredity status was defined by the presence of clinical features associated with well-known familial syndromes (medullary thyroid carcinoma, hyperparathyroidism, hemangioblastomas of retina and/or central nervous system, renal cell carcinoma, neurofibromas, café-au-lait spots, and head and neck paragangliomas) or diagnosis of pheochromocytoma and/or paraganglioma in at least one first-degree relative. In all, 76 catecholamine-secreting pheochromocytomas or paragangliomas representing well-characterized hereditary variants cited above, familial tumors of undetermined genetic cause, and sporadic tumors were included in this study (see Dataset S1). Of these, 15 samples belonged to seven different families presenting with bilateral tumors and/or familial history of recurrent pheochromocytoma and/or paraganglioma. No additional clinical features associated with hereditary pheochromocytoma were identified in these individuals. Of the seventy-six tumors, 13 were located outside the adrenal gland (one was mediastinal, one retrocardiac, and the remaining 11 were in periaortic or perirenal locations). No head or neck paragangliomas or tumors with *SDHC* mutations were included in this series.

#### RNA isolation and microarray preparation.

Total RNA was extracted from each frozen tumor specimen, and biotinylated cRNAs were generated using Trizol (Invitrogen, Carlsbad, California, United States) according to the manufacturer's instructions. Eighty-four tumor samples (including six replicates) were hybridized overnight to U133A oligonucleotide microarrays (Affymetrix, Santa Clara, California, United States), which included approximately 22,000 probe sets. In four duplicate cases two aliquots of RNA were separately used for target preparation and subsequent analysis, and in two cases, the same source cRNA was used in two independent hybridizations. Arrays were subsequently developed with phycoerythrin-conjugated streptavidin (SAPE) and biotinylated anti-streptavidin, and scanned to obtain quantitative gene expression levels. The raw gene expression values were scaled to account for differences in global chip intensity using MAS software (Affymetrix). Four scans (two duplicates and two unique tumors) were excluded because of poor quality. In total, 76 unique tumor samples were used for the analysis.

#### Normalization and model-based expression analysis.

All arrays were normalized using dChip software v1.3 [28]. Model-based expression index was obtained by PM/MM difference algorithm in dChip. Gene filtering and hierarchical clustering analysis were also performed in dChip [29]. To deal with variable degrees of quality in sample hybridization and resulting control parameters, such as absolute (%P) call and 5′/3′ ratios of housekeeping genes (GAPDH and actin), we subclassified the samples into three categories: superior, good, and satisfactory quality. For each gene, samples belonging to each category were separately computed for coefficient of variation (standard deviation/mean) for gene filtering and standardized (to achieve mean = 0 and SD = 1) for gene and sample clustering. Duplicated samples were combined prior to standardization.

#### Unsupervised clustering analysis.

Samples were clustered using an unsupervised hierarchical clustering method to delineate groups with biological distinction. Filtering parameters were set to define the genes that showed the highest variation among the sample set. A selection was made of 508 genes using the following parameters: 0.6 < average SD/mean across categories < 10, and having a mean intensity value of >100 units in at least 20% of samples.

The reliability of the clusters was verified by a resampling method based on the standard errors for expression values [30]. This subsampling procedure was repeated 100 times to refine the cluster parameters. The most stable clusters resulting from multiple iterations were defined and used for subsequent analysis.

#### Supervised analysis.

Once the main clusters were defined by the method above, tumors that represented individual genetic classes were used for supervised analysis: MEN2 versus VHL pheochromocytomas, or MEN2 versus combined SDHB and SDHD (SDH) samples. The strength of gene expression differences between each pair of classes (defined as the “training set”) was assessed using two supervised machine learning algorithms, *k* nearest neighbor and weighted-voting, as previously described [31,32]. Duplicate samples were also included in this analysis. Data were preprocessed by applying thresholds of ten minimum and 16,000 maximum genes, which then were filtered by requiring a 5-fold minimum variation and 50 minimum absolute difference. Models were evaluated using leave-one-out cross-validation. The differential genes were reselected after each sample withdrawal. Probes (features) to be used in the models were selected by ranking the genes according to the signal-to-noise metric [31]. After the number of probes was selected to find the minimum error rate, a model was trained using data for the pair of tumor classes and tested on data for the remaining samples (defined as the “test set”). Prediction performance on the test samples was used to confirm the similarity of sample types.

#### Pathway analysis.

To gather insights into the function of genes associated with the clusters defined by the comparisons described above, we used a statistical method designed to test for the enrichment of groups of genes in data generated from expression studies, GSEA [33]. GSEA considers predefined gene sets representing pathways of interest and determines whether the members of these sets are overrepresented in a list of genes that has been ordered by their correlation with a specific phenotype or class distinction.

The output of GSEA is a normalized enrichment score (NES) that represents a measure of the degree of enrichment of the gene set at the top (highly correlated) or bottom (anti-correlated) of the ordered gene list. The NES is used to produce a *p*-value that measures the significance of that score. This is obtained by permutation testing, which involves shuffling the class template associated with the data to determine how often an observed NES occurs by chance. *p*-Values are adjusted to account for multiple hypothesis testing [34]. The gene sets used for analysis in the current study were obtained from Gene Ontology (www.geneontology.org), GenMAPP (www.genmapp.org), and Biocarta (www.biocarta.com); manually curated proteome datasets were also used [34–36].

#### DNA sequencing.

Direct sequencing of familial samples and 20 sporadic tumors was performed from tumor and germline DNA, whenever available, using PCR products that encompass exons and intron–exon boundaries of *RET* (exons 10, 11, and 13–16), *VHL* (exons 1–3), *SDHD* (exons 1–4), and *SDHB* (exons 1–8), as previously described [7,37,38]. *SDHC* was also sequenced in familial tumors with no detectable mutation [7].

#### Western blots and transfections.

Whole cell lysates from tumors and normal adrenal medullas were prepared as previously described [39], and 50 μg was run on 12% SDS gels, transferred to PVDF membranes and hybridized with antibodies against SDHB and SDHA (Molecular Probes, Eugene, Oregon, United States), RET (Immuno-Biological Laboratories, Gunma, Japan), or β-actin (Sigma, St. Louis, Missouri, United States), according to the manufacturers' instructions. Filters were developed with a chemiluminescence assay (Pierce Biotechnology, Rockford, Illinois, United States) and images captured using the VersaDoc Imaging system (Bio-Rad, Hercules, California, United States).

The MPC 9/3L cell lines derived from NF1^±^ mice were cultured as described [40]. HEK293 cells were cultured in DMEM and 10% fetal bovine serum supplemented with 100 U/ml penicillin and 100 μg/ml streptomycin. HIF1α expression was induced in MPC 9/3L or HEK293 cells by treatment with 150 μM cobalt chloride, which blocks prolyl hydroxylation of HIF1α and its binding to VHL [41], for the indicated times (see Figure 4A). A HIF1α double mutant (P402A/P564A) that is resistant to VHL-mediated proteasome degradation [42] was generated by site-directed mutagenesis (Quick Change, Stratagene, La Jolla, California, United States) and cloned into p3X-FLAG vector (Sigma). HEK293 cells were transfected with the HIF1α P402A/P564A double mutant or an empty vector using Lipofectamine 2000, as recommended by the manufacturer (Invitrogen). Transfected cells were harvested at 48 h and assayed by Western blotting, as above. Membranes were probed with the following antibodies: SDHB, as described above, HIF1α (BD Biosciences, San Jose, California, United States), Glut1, used as a surrogate for HIF1α activity (Alpha Diagnostic, San Antonio, Texas, United States), and FLAG (Sigma). β-actin was used as a loading control, as above. The lentiviral shRNA expression vector FSIPPW was used, as previously described [43]. The shRNA expression construct targeting HIF1α (FSIPPW-HIF) is directed against the sequence 5′-AACTAACTGGACACAGTGTGTTT-3′, which is conserved in mouse, rat, and human HIF1α. FSIPPW-eGFP, packaging of lentiviruses, and infection of cell lines were performed as previously described [43]. A2058 melanoma cells stably expressing HIF1α shRNA (FSIPPW-HIF) or control pEGFP shRNA (FSIPPW-EGFP) were cultured in DMEM, 10% FBS, and 2 μg/ml puromycin. Infected cell lines were selected with 2 μg/ml puromycin (Sigma). Cells were exposed to 150 μM cobalt chloride for 24 or 48 h, and lysates obtained as above.

#### Quantitative Real-Time PCR.

Quantitative real-time PCR was performed in cDNA from 20 tumors (ten from Cluster 1 and ten from Cluster 2) from the cohort above using the iCycler iQ Real-Time PCR Detection System (Bio-Rad). SYBR green fluorescence (Bio-Rad) was used for quantification, according to the manufacturer's instructions. Primer sequences and PCR conditions are available upon request.

#### Immunohistochemistry.

Immunohistochemical analysis was performed on 4-μm-thin sections of formalin-fixed tissue obtained from the archives of Brigham and Women's Hospital and from consultation. Clinical data and additional follow-up information were provided by the referring clinician and/or pathologists. Slides were processed according to standard protocol using primary antibodies SDHB (1:1,000 dilution, Molecular Probes) and the Envision Plus Detection System (Dako, Carpinteria, California, United States) for antigen–antibody detection. Heart muscle and normal adrenal tissue were included as positive controls. Negative controls (no primary antibody) were also maintained throughout. Immunoreactivity was graded semi-quantitatively using the following scale: 0, no staining; 1+, <5% of tumor cells reactive; 2+, 5%–25% of tumor cells reactive; 3+, 25%–50% of tumor cells reactive; 4+, >50% of tumor cells reactive (weak intensity); 5+, >50% of tumor cells reactive (moderate intensity); and 6+, >50% of tumor cells reactive (strong intensity).

## Supporting Information

Dataset S1 Summary of Clinical Data and Classification of Pheochromocytomas by Genetic Groups (13 KB PDF)

Dataset S2 Filtered Gene List from the Unsupervised Analysis (137 KB PDF)

Dataset S3 Results of Real-Time PCR and Western Blot Analyses of Differentially Expressed Genes (27 KB PDF)

Dataset S4 Cross-Validation and Gene Set Analysis Results (Gene Lists and Heat Map Images) of Two-Class Comparisons by Supervised Learning Methods (345 KB PDF)

Dataset S5 Expression of Mitochondrial Complex II Subunits in Pheochromocytomas by Cluster Distribution (12 KB PDF)

## Accession Numbers

The data discussed in this publication have been deposited in NCBI's Gene Expression Omnibus (GEO, http://www.ncbi.nlm.nih.gov/geo/) and are accessible through GEO Series accession number GSE2841.

## Abbreviations

GSEA: gene set enrichment analysis
HIF: hypoxia-inducible factor
HIF1α: hypoxia-inducible factor 1 subunit α
MEN2: multiple endocrine neoplasia type 2
MPC 9/3L: mouse pheochromocytoma cell line 9/3L
NES: normalized enrichment score
NF1: neurofibromatosis type 1
PGL[number]: paraganglioma syndrome type [number]
SDH: succinate dehydrogenase
shRNA: short hairpin RNA
VHL: von Hippel-Lindau disease
