# Supplementary material for: A HIF1α Regulatory Loop Links Hypoxia and Mitochondrial Signals in Pheochromocytomas
Source: PLoS Genet. 2005 Jul 25;1(1):e8. doi: 10.1371/journal.pgen.0010008 (PMC1183527; doi:10.1371/journal.pgen.0010008)
Supplement: Dataset S1 — (13 KB PDF) [file pgen.0010008.sd001.pdf]

Suppl. Data 1. Summary of clinical data and classification of pheochromocytomas by genetic groups.

Age		4-78y
Gender		48 female<br>24 male
Tumor location		63 adrenal<br>13 extra-adrenal
Malignant tumors*		2
Sporadic tumors		37
Familial tumors		39
	FP1	4
	FP2	3
	FP3	3
	FP4	2
	FP5	1
	FP6	1
	FP7	1
	MEN 2A	10[a]
	NF1	2[b]
	SDHB	5[c]
	SDHD	1
	VHL	6[d]

[*] defined by the presence of distant metastases; Hereditary samples represent variable numbers of independent families in this cohort: [a] MEN 2A tumors, five families; [b] NF1 tumors, two families; [c] SDHB tumors, five families; and [d] VHL tumors, three families; FP1-7 represent 7 independent families or individuals with familial history of pheochromocytoma without an identifiable mutation.
