# Supplementary material for: A HIF1α Regulatory Loop Links Hypoxia and Mitochondrial Signals in Pheochromocytomas
Source: PLoS Genet. 2005 Jul 25;1(1):e8. doi: 10.1371/journal.pgen.0010008 (PMC1183527; doi:10.1371/journal.pgen.0010008)
Supplement: Dataset S2 — (137 KB PDF) [file pgen.0010008.sd002.pdf]

Suppl. Data 2. Filtered Gene List-Unsupervised Hierarchical Clustering of Pheochromocytomas
[FILTERING_CRITERIA]
Variation across samples: 0.65 < Standard deviation / Mean < 10.00
P call % in the array used >= 20%

probe set	gene	Accession	LocusLink
200024_at	ribosomal protein S5	NM_001009.1	6193
200056_s_at	nuclear DNA-binding protein	NM_006333.1	10438
200096_s_at	ATPase, H+ transporting, lysosomal 9kDa, V0 subunit e	AI862255	8992
200097_s_at	heterogeneous nuclear ribonucleoprotein K	AI701949	3190
200605_s_at	protein kinase, cAMP-dependent, regulatory, type I, alpha (tissue specific extinguisher 1)	NM_002734.1	5573
200606_at	desmoplakin (DPI, DPII)	NM_004415.1	1832
200650_s_at	lactate dehydrogenase A	NM_005566.1	3939
200654_at	procollagen-proline, 2-oxoglutarate 4-dioxygenase (proline 4-hydroxylase), beta polypept	J02783.1	5034
200656_s_at	procollagen-proline, 2-oxoglutarate 4-dioxygenase (proline 4-hydroxylase), beta polypept	NM_000918.1	5034
200662_s_at	translocase of outer mitochondrial membrane 20 (yeast) homolog	NM_014765.1	9804
200665_s_at	secreted protein, acidic, cysteine-rich (osteonectin)	NM_003118.1	6678
200672_x_at	spectrin, beta, non-erythrocytic 1	NM_003128.1	6711
200750_s_at	RAN, member RAS oncogene family	AF054183.1	5901
200764_s_at	catenin (cadherin-associated protein), alpha 1 (102kDa	AI826881	1495
200782_at	annexin A5	NM_001154.2	308
200783_s_at	stathmin 1/oncoprotein 18	NM_005563.2	3925
200800_s_at	heat shock 70kDa protein 1A	NM_005345.3	3303
200832_s_at	stearoyl-CoA desaturase (delta-9-desaturase)	AB032261.1	6319
200853_at	H2A histone family, member Z	NM_002106.1	3015
200862_at	24-dehydrocholesterol reductase	NM_014762.1	1718
200866_s_at	prosaposin (variant Gaucher disease and variant metachromatic leukodystrophy)	M32221.1	5660
200883_at	ubiquinol-cytochrome c reductase core protein II	NM_003366.1	7385
200884_at	creatine kinase, brain	NM_001823.1	1152
200968_s_at	peptidylprolyl isomerase B (cyclophilin B)	NM_000942.1	5479
200973_s_at	tetraspan 3	NM_005724.1	10099
200974_at	actin, alpha 2, smooth muscle, aorta	NM_001613.1	59
201005_at	CD9 antigen (p24)	NM_001769.1	928
201013_s_at	phosphoribosylaminoimidazole carboxylase, phosphoribosylaminoimidazole succinocarb	AA902652	10606
201020_at	tyrosine 3-monooxygenase/tryptophan 5-monooxygenase activation protein, eta polypept	NM_003405.1	7533
201050_at	similar to vaccinia virus HindIII K4L ORF	NM_012268.1	23646
201058_s_at	myosin, light polypeptide 9, regulatory	NM_006097.1	10398
201078_at	transmembrane 9 superfamily member 2	NM_004800.1	9375
201082_s_at	dynactin 1 (p150, glued homolog, Drosophila)	NM_004082.2	1639
201110_s_at	thrombospondin 1	NM_003246.1	7057
201117_s_at	carboxypeptidase E	NM_001873.1	1363
201132_at	heterogeneous nuclear ribonucleoprotein H2 (H')	NM_019597.1	3188
201162_at	insulin-like growth factor binding protein 7	NM_001553.1	3490
201163_s_at	insulin-like growth factor binding protein 7	NM_001553.1	3490
201229_s_at	ariadne homolog 2 (Drosophila)	BC000422.1	10425
201242_s_at	ATPase, Na+/K+ transporting, beta 1 polypeptide	BC000006.1	481
201243_s_at	ATPase, Na+/K+ transporting, beta 1 polypeptide	NM_001677.1	481
201272_at	aldo-keto reductase family 1, member B1 (aldose reductase)	NM_001628.1	231
201280_s_at	disabled homolog 2, mitogen-responsive phosphoprotein (Drosophila)	NM_001343.1	1601
201315_x_at	interferon induced transmembrane protein 2 (1-8D)	NM_006435.1	10581
201324_at	epithelial membrane protein 1	NM_001423.1	2012
201348_at	glutathione peroxidase 3 (plasma)	NM_002084.2	2878

201369_s_at	zinc finger protein 36, C3H type-like 2	NM_006887.1	678
201387_s_at	ubiquitin carboxyl-terminal esterase L1 (ubiquitin thiolesterase)	NM_004181.1	7345
201416_at	SRY (sex determining region Y)-box 4	NM_003107.1	6659
201426_s_at	vimentin	AI922599	7431
201432_at	catalase	NM_001752.1	847
201465_s_at	v-jun sarcoma virus 17 oncogene homolog (avian)	BC002646.1	3725
201496_x_at	myosin, heavy polypeptide 11, smooth muscle	AI889739	4629
201497_x_at	myosin, heavy polypeptide 11, smooth muscle	NM_022844.1	4629
201505_at	laminin, beta 1	NM_002291.1	3912
201552_at	lysosomal-associated membrane protein 1	NM_005561.2	3916
201578_at	podocalyxin-like	NM_005397.1	5420
201601_x_at	interferon induced transmembrane protein 1 (9-27)	NM_003641.1	8519
201619_at	peroxiredoxin 3	NM_006793.1	10935
201630_s_at	acid phosphatase 1, soluble	NM_004300.1	52
201650_at	keratin 19	NM_002276.1	3880
201667_at	gap junction protein, alpha 1, 43kDa (connexin 43)	NM_000165.2	2697
201669_s_at	myristoylated alanine-rich protein kinase C substrate	NM_002356.4	4082
201693_s_at	early growth response 1	AV733950	1958
201694_s_at	early growth response 1	NM_001964.1	1958
201744_s_at	lumican	NM_002345.1	4060
201761_at	methylene tetrahydrofolate dehydrogenase (NAD+ dependent), methenyltetrahydrofolate	NM_006636.2	10797
201839_s_at	tumor-associated calcium signal transducer 1	NM_002354.1	4072
201841_s_at	heat shock 27kDa protein 1	NM_001540.2	3315
201852_x_at	collagen, type III, alpha 1 (Ehlers-Danlos syndrome type IV, autosomal dominant)	AI813758	1281
201858_s_at	proteoglycan 1, secretory granule	J03223.1	5552
201860_s_at	plasminogen activator, tissue	NM_000930.1	5327
201867_s_at	transducin (beta)-like 1X-linked	NM_005647.1	6907
201893_x_at	decorin	AF138300.1	1634
201909_at	ribosomal protein S4, Y-linked	NM_001008.1	6192
201923_at	peroxiredoxin 4	NM_006406.1	10549
201951_at	activated leukocyte cell adhesion molecule	NM_001627.1	214
202015_x_at	methionyl aminopeptidase 2	NM_006838.1	10988
202016_at	mesoderm specific transcript homolog (mouse)	NM_002402.1	4232
202035_s_at	secreted frizzled-related protein 1	AI332407	6422
202054_s_at	aldehyde dehydrogenase 3 family, member A2	NM_000382.1	224
202149_at	enhancer of filamentation 1 (cas-like docking; Crk-associated substrate related)	AL136139	10543
202196_s_at	dickkopf homolog 3 (Xenopus laevis)	NM_013253.1	27122
202232_s_at	dendritic cell protein	NM_006360.1	10480
202237_at	nicotinamide N-methyltransferase	NM_006169.1	4837
202241_at	phosphoprotein regulated by mitogenic pathways	NM_025195.1	10221
202243_s_at	proteasome (prosome, macropain) subunit, beta type, 4	NM_002796.1	5692
202340_x_at	nuclear receptor subfamily 4, group A, member 1	NM_002135.1	3164
202363_at	sparc/osteonectin, cwcv and kazal-like domains proteoglycan (testican)	AF231124.1	6695
202404_s_at	collagen, type I, alpha 2	NM_000089.1	1278
202409_at	Homo sapiens cDNA: FLJ22066 fis, clone HEP10611	X07868	
202410_x_at	insulin-like growth factor 2 (somatomedin A)	NM_000612.2	3481
202411_at	interferon, alpha-inducible protein 27	NM_005532.1	3429
202422_s_at	fatty-acid-Coenzyme A ligase, long-chain 4	NM_022977.1	2182
202436_s_at	cytochrome P450, subfamily I (dioxin-inducible), polypeptide 1 (glaucoma 3, primary infar	NM_000104.2	1545
202508_s_at	synaptosomal-associated protein, 25kDa	NM_003081.1	6616
202524_s_at	KIAA0275 gene product	NM_014767.1	9806
202551_s_at	cysteine-rich motor neuron 1	BG546884	51232

202552_s_at	cysteine-rich motor neuron 1	NM_016441.1	51232
202591_s_at	single-stranded DNA binding protein	NM_003143.1	6742
202601_s_at	HIV TAT specific factor 1	AI373539	27336
202602_s_at	HIV TAT specific factor 1	NM_014500.1	27336
202635_s_at	polymerase (RNA) II (DNA directed) polypeptide K, 7.0kDa	NM_005034.1	5440
202672_s_at	activating transcription factor 3	NM_001674.1	467
202718_at	insulin-like growth factor binding protein 2, 36kDa	NM_000597.1	3485
202768_at	FBJ murine osteosarcoma viral oncogene homolog B	NM_006732.1	2354
202859_x_at	interleukin 8	NM_000584.1	3576
202917_s_at	S100 calcium binding protein A8 (calgranulin A)	NM_002964.2	6279
202935_s_at	SRY (sex determining region Y)-box 9 (campomelic dysplasia, autosomal sex-reversal)	AI382146	6662
202948_at	interleukin 1 receptor, type I	NM_000877.1	3554
202992_at	complement component 7	NM_000587.1	730
203000_at	EST	BF967657	
203001_s_at	stathmin-like 2	NM_007029.1	11075
203029_s_at	protein tyrosine phosphatase, receptor type, N polypeptide 2	NM_002847.1	5799
203060_s_at	3'-phosphoadenosine 5'-phosphosulfate synthase 2	AF074331.1	9060
203132_at	retinoblastoma 1 (including osteosarcoma)	NM_000321.1	5925
203180_at	aldehyde dehydrogenase 1 family, member A3	NM_000693.1	220
203243_s_at	LIM protein (similar to rat protein kinase C-binding enigma)	NM_006457.1	10611
203254_s_at	talin 1	NM_006289.1	7094
203382_s_at	apolipoprotein E	NM_000041.1	348
203392_s_at	C-terminal binding protein 1	NM_001328.1	1487
203413_at	NEL-like 2 (chicken)	NM_006159.1	4753
203485_at	reticulon 1	NM_021136.1	6252
203607_at	Sac domain-containing inositol phosphatase 2	NM_014937.1	22876
203619_s_at	lifeguard	NM_012306.1	23017
203628_at	insulin-like growth factor 1 receptor	NM_000875.2	3480
203646_at	ferredoxin 1	NM_004109.2	2230
203647_s_at	ferredoxin 1	M18003.1	2230
203803_at	prenylcysteine lyase	N45309	51449
203860_at	propionyl Coenzyme A carboxylase, alpha polypeptide	NM_000282.1	5095
203889_at	secretory granule, neuroendocrine protein 1 (7B2 protein)	NM_003020.1	6447
203896_s_at	phospholipase C, beta 4	NM_000933.1	5332
203934_at	kinase insert domain receptor (a type III receptor tyrosine kinase)	NM_002253.1	3791
203961_at	nebulette	AL157398	10529
203962_s_at	nebulette	NM_006393.1	10529
203980_at	fatty acid binding protein 4, adipocyte	NM_001442.1	2167
203998_s_at	synaptotagmin I	NM_005639.1	6857
203999_at	synaptotagmin I	NM_005639.1	6857
204014_at	dual specificity phosphatase 4	NM_001394.2	1846
204018_x_at	hemoglobin, alpha 1	NM_000558.2	3039
204035_at	secretogranin II (chromogranin C)	NM_003469.2	7857
204071_s_at	tumor protein p53-binding protein	NM_005802.1	10210
204081_at	neurogranin (protein kinase C substrate, RC3)	NM_006176.1	4900
204102_s_at	eukaryotic translation elongation factor 2	NM_001961.1	1938
204120_s_at	adenosine kinase	NM_001123.1	132
204121_at	growth arrest and DNA-damage-inducible, gamma	NM_006705.2	10912
204141_at	tubulin, beta polypeptide	NM_001069.1	7280
204151_x_at	aldo-keto reductase family 1, member C1 (dihydrodiol dehydrogenase 1; 20-alpha (3-alph	NM_001353.2	1645
204224_s_at	GTP cyclohydrolase 1 (dopa-responsive dystonia)	NM_000161.1	2643
204260_at	chromogranin B (secretogranin 1)	NM_001819.1	1114

204273_at	endothelin receptor type B	NM_000115.1	1910
204337_at	regulator of G-protein signalling 4	AL514445	5999
204400_at	signal transduction protein (SH3 containing)	NM_005864.1	10278
204409_s_at	eukaryotic translation initiation factor 1A, Y chromosome	BC005248.1	9086
204465_s_at	internexin neuronal intermediate filament protein, alpha	NM_004692.1	9118
204472_at	GTP binding protein overexpressed in skeletal muscle	NM_005261.1	2669
204557_s_at	zinc-finger protein DZIP1	NM_014934.1	22873
204597_x_at	stanniocalcin 1	NM_003155.1	6781
204621_s_at	nuclear receptor subfamily 4, group A, member 2	AI935096	4929
204622_x_at	nuclear receptor subfamily 4, group A, member 2	NM_006186.1	4929
204665_at	hypothetical protein FLJ21168	NM_025073.1	80143
204675_at	steroid-5-alpha-reductase, alpha polypeptide 1 (3-oxo-5 alpha-steroid delta 4-dehydroger	NM_001047.1	6715
204697_s_at	chromogranin A (parathyroid secretory protein 1)	NM_001275.2	1113
204719_at	ATP-binding cassette, sub-family A (ABC1), member 8	NM_007168.1	10351
204723_at	voltage-gated sodium channel beta-3 subunit (scn3b gene)	NM_018400.1	55800
204743_at	neuronal protein	NM_013259.1	29114
204748_at	prostaglandin-endoperoxide synthase 2 (prostaglandin G/H synthase and cyclooxygenas	NM_000963.1	5743
204850_s_at	doublecortex; lissencephaly, X-linked (doublecortin)	NM_000555.1	1641
204869_at	proprotein convertase subtilisin/kexin type 2	AL031664	5126
204870_s_at	proprotein convertase subtilisin/kexin type 2	NM_002594.1	5126
204897_at	prostaglandin E receptor 4 (subtype EP4)	NM_000958.1	5734
204916_at	receptor (calcitonin) activity modifying protein 1	NM_005855.1	10267
205000_at	DEAD/H (Asp-Glu-Ala-Asp/His) box polypeptide, Y chromosome	NM_004660.2	8653
205013_s_at	adenosine A2a receptor	NM_000675.2	135
205048_s_at	phosphoserine phosphatase-like	NM_003832.1	8781
205083_at	aldehyde oxidase 1	NM_001159.2	316
205109_s_at	Rho guanine nucleotide exchange factor (GEF) 4	NM_015320.1	50649
205113_at	neurofilament 3 (150kDa medium)	NM_005382.1	4741
205114_s_at	chemokine (C-C motif) ligand 3	NM_002983.1	6348
205187_at	MAD, mothers against decapentaplegic homolog 5 (Drosophila)	AF010601.1	4090
205206_at	Kallmann syndrome 1 sequence	NM_000216.1	3730
205278_at	glutamate decarboxylase 1 (brain, 67kDa)	NM_000817.1	2571
205280_at	glycine receptor, beta	NM_000824.1	2743
205281_s_at	phosphatidylinositol glycan, class A (paroxysmal nocturnal hemoglobinuria)	NM_002641.1	5277
205292_s_at	heterogeneous nuclear ribonucleoprotein A2/B1	NM_002137.1	3181
205311_at	dopa decarboxylase (aromatic L-amino acid decarboxylase)	NM_000790.1	1644
205352_at	serine (or cysteine) proteinase inhibitor, clade I (neuroserpin), member 1	NM_005025.1	5274
205358_at	glutamate receptor, ionotropic, AMPA 2	NM_000826.1	2891
205366_s_at	homeo box B6	NM_018952.1	3216
205413_at	chromosome 11 open reading frame 8	NM_001584.1	744
205433_at	butyrylcholinesterase	NM_000055.1	590
205483_s_at	interferon-stimulated protein, 15 kDa	NM_005101.1	9636
205586_x_at	VGF nerve growth factor inducible	NM_003378.1	7425
205633_s_at	aminolevulinate, delta-, synthase 1	NM_000688.1	211
205728_at	odz, odd Oz/ten-m homolog 1(Drosophila)	AL022718	10178
205795_at	neurexin 3	NM_004796.1	9369
205825_at	proprotein convertase subtilisin/kexin type 1	NM_000439.2	5122
205830_at	calmegin	NM_004362.1	1047
205857_at	solute carrier family 18 (vesicular monoamine), member 2	AI269290	6571
205923_at	reelin	NM_005045.1	5649
205952_at	potassium channel, subfamily K, member 3	NM_002246.1	3777
205978_at	klotho	NM_004795.1	9365

206001_at	neuropeptide Y	NM_000905.1	4852
206042_x_at	SNRPN upstream reading frame	NM_022804.1	8926
206080_at	KIAA0450 gene product	NM_014638.1	9651
206104_at	ISL1 transcription factor, LIM/homeodomain, (islet-1)	NM_002202.1	3670
206115_at	early growth response 3	NM_004430.1	1960
206162_x_at	synaptotagmin V	NM_003180.1	6861
206163_at	mab-21-like 1 (C. elegans)	NM_005584.1	4081
206172_at	interleukin 13 receptor, alpha 2	NM_000640.1	3598
206339_at	cocaine- and amphetamine-regulated transcript	NM_004291.1	9607
206434_at	testican 3	NM_016950.1	50859
206450_at	dopamine beta-hydroxylase (dopamine beta-monooxygenase)	NM_000787.1	1621
206478_at	KIAA0125 gene product	NM_014792.1	9834
206502_s_at	insulinoma-associated 1	NM_002196.1	3642
206552_s_at	tachykinin, precursor 1 (substance K, substance P, neurokinin 1, neurokinin 2, neuromed	NM_003182.1	6863
206598_at	insulin	NM_000207.1	3630
206637_at	G protein-coupled receptor 105	NM_014879.1	9934
206698_at	Kell blood group precursor (McLeod phenotype)	NM_021083.1	7504
206793_at	phenylethanolamine N-methyltransferase	NM_002686.1	5409
207002_s_at	pleiomorphic adenoma gene-like 1	NM_002656.1	5325
207009_at	paired mesoderm homeobox 2b	NM_003924.2	8929
207076_s_at	argininosuccinate synthetase	NM_000050.1	445
207447_s_at	UDP-N-acetylglucosamine:a-1,3-D-mannoside beta-1,4-N-acetylglucosaminyltransferase	NM_013244.1	25834
207695_s_at	immunoglobulin superfamily, member 1	NM_001555.1	3547
207768_at	early growth response 4	NM_001965.1	1961
207974_s_at	S-phase kinase-associated protein 1A (p19A)	NM_006930.1	6500
207980_s_at	Cbp/p300-interacting transactivator, with Glu/Asp-rich carboxy-terminal domain, 2	NM_006079.1	10370
208291_s_at	tyrosine hydroxylase	NM_000360.1	7054
208308_s_at	glucose phosphate isomerase	NM_000175.1	2821
208319_s_at	RNA binding motif protein 3	NM_006743.1	5935
208451_s_at	complement component 4B	NM_000592.2	721
208517_x_at	basic transcription factor 3	NM_001207.1	689
208605_s_at	neurotrophic tyrosine kinase, receptor, type 1	NM_002529.2	4914
208623_s_at	villin 2 (ezrin)	J05021.1	7430
208650_s_at	CD24 antigen (small cell lung carcinoma cluster 4 antigen)	BG327863	934
208651_x_at	CD24 antigen (small cell lung carcinoma cluster 4 antigen)	M58664.1	934
208669_s_at	CREBBP/EP300 inhibitory protein 1	AF109873.1	23741
208671_at	KIAA1253 protein	AF164794.1	57515
208687_x_at	heat shock 70kDa protein 8	AF352832.1	3312
208689_s_at	ribophorin II	BC003560.1	6185
208729_x_at	major histocompatibility complex, class I, B	D83043.1	3106
208743_s_at	tyrosine 3-monooxygenase/tryptophan 5-monooxygenase activation protein, beta polypep	BC001359.1	7529
208791_at	clusterin (complement lysis inhibitor, SP-40,40, sulfated glycoprotein 2, testosterone-repr	M25915.1	1191
208792_s_at	clusterin (complement lysis inhibitor, SP-40,40, sulfated glycoprotein 2, testosterone-repr	M25915.1	1191
208810_at	similar to MRJ gene for a member of the DNAJ protein family (H. sapiens)	AF080569.1	136442
208850_s_at	Thy-1 cell surface antigen	AL558479	7070
208892_s_at	dual specificity phosphatase 6	BC003143.1	1848
208944_at	transforming growth factor, beta receptor II (70/80kDa)	D50683.1	7048
209004_s_at	F-box and leucine-rich repeat protein 5	AF142481.1	26234
209070_s_at	regulator of G-protein signalling 5	AI183997	8490
209101_at	connective tissue growth factor	M92934.1	1490
209116_x_at	hemoglobin, beta	M25079.1	3043
209138_x_at	immunoglobulin lambda locus	M87790.1	3535

209140_x_at	major histocompatibility complex, class I, B	L42024.1	3106
209147_s_at	phosphatidic acid phosphatase type 2A	AB000888.1	8611
209160_at	aldo-keto reductase family 1, member C3 (3-alpha hydroxysteroid dehydrogenase, type II	AB018580.1	8644
209163_at	cytochrome b-561	AL514271	1534
209164_s_at	cytochrome b-561	BC002976.1	1534
209183_s_at	decidual protein induced by progesterone	AL136653.1	11067
209189_at	v-fos FBJ murine osteosarcoma viral oncogene homolog	BC004490.1	2353
209243_s_at	zinc finger, imprinted 2	AF208967.1	23619
209278_s_at	tissue factor pathway inhibitor 2	L27624.1	7980
209318_x_at	pleiomorphic adenoma gene-like 1	BG547855	5325
209328_x_at	hypothetical protein HSPC111	BC000587.1	51491
209348_s_at	v-maf musculoaponeurotic fibrosarcoma oncogene homolog (avian)	AF055376.1	4094
209355_s_at	phosphatidic acid phosphatase type 2B	AB000889.1	8613
209357_at	Cbp/p300-interacting transactivator, with Glu/Asp-rich carboxy-terminal domain, 2	AF109161.1	10370
209443_at	serine (or cysteine) proteinase inhibitor, clade A (alpha-1 antiproteinase, antitrypsin), mer	J02639.1	5104
209458_x_at	hemoglobin, alpha 1	AF105974.1	3039
209459_s_at	NPD009 protein	AF237813.1	57416
209460_at	NPD009 protein	AF237813.1	57416
209462_at	amyloid beta (A4) precursor-like protein 1	U48437.1	333
209465_x_at	pleiotrophin (heparin binding growth factor 8, neurite growth-promoting factor 1)	AL565812	5764
209466_x_at	pleiotrophin (heparin binding growth factor 8, neurite growth-promoting factor 1)	M57399.1	5764
209496_at	retinoic acid receptor responder (tazarotene induced) 2	BC000069.1	5919
209570_s_at	DNA segment on chromosome 4 (unique) 234 expressed sequence	BC001745.1	27065
209598_at	paraneoplastic antigen MA2	AB020690.1	10687
209602_s_at	GATA binding protein 3	AI796169	2625
209613_s_at	alcohol dehydrogenase IB (class I), beta polypeptide	M21692.1	125
209699_x_at	aldo-keto reductase family 1, member C2 (dihydrodiol dehydrogenase 2; bile acid binding	U05598.1	1646
209710_at	hypothetical protein MGC2306	AL563460	84724
209771_x_at	CD24 antigen (small cell lung carcinoma cluster 4 antigen)	AA761181	934
209803_s_at	tumor suppressing subtransferable candidate 3	AF001294.1	7262
209846_s_at	butyrophilin, subfamily 3, member A2	BC002832.1	11118
209875_s_at	secreted phosphoprotein 1 (osteopontin, bone sialoprotein I, early T-lymphocyte activatio	M83248.1	6696
209883_at	chromosome 1 open reading frame 17	AF288389.1	23127
209956_s_at	calcium/calmodulin-dependent protein kinase (CaM kinase) II beta	U23460.1	816
209987_s_at	achaete-scute complex-like 1 (Drosophila)	BC002341.1	429
209988_s_at	achaete-scute complex-like 1 (Drosophila)	BC001638.1	429
209993_at	ATP-binding cassette, sub-family B (MDR/TAP), member 1	AF016535.1	5243
209994_s_at	ATP-binding cassette, sub-family B (MDR/TAP), member 1	AF016535.1	5243
210078_s_at	potassium voltage-gated channel, shaker-related subfamily, beta member 1	L39833.1	7881
210090_at	activity-regulated cytoskeleton-associated protein	AF193421.1	23237
210095_s_at	insulin-like growth factor binding protein 3	M31159.1	3486
210221_at	cholinergic receptor, nicotinic, alpha polypeptide 3	BC000513.1	1136
210302_s_at	mab-21-like 2 (C. elegans)	AF262032.1	10586
210338_s_at	heat shock 70kDa protein 8	AB034951.1	3312
210353_s_at	solute carrier family 6 (neurotransmitter transporter, noradrenalin), member 2	M65105.1	6530
210358_x_at	hypothetical protein MGC2306	BC002557.1	84724
210404_x_at	calcium/calmodulin-dependent protein kinase (CaM kinase) II beta	AF078803.1	816
210414_at	fibronectin leucine rich transmembrane protein 1	AF169675.1	23769
210445_at	fatty acid binding protein 6, ileal (gastrotropin)	U19869.1	2172
210495_x_at	fibronectin 1	AF130095.1	2335
210514_x_at	HLA-G histocompatibility antigen, class I, G	AF226990.2	3135
210764_s_at	cysteine-rich, angiogenic inducer, 61	AF003114.1	3491

210881_s_at	insulin-like growth factor 2 (somatomedin A)	M17863.1	3481
211161_s_at	gb:AF130082.1 /DEF=Homo sapiens clone FLC1492 PRO3121 mRNA, complete cds. /FI	AF130082.1	
211276_at	my048 protein	AF063606.1	140597
211421_s_at	ret proto-oncogene (multiple endocrine neoplasia and medullary thyroid carcinoma 1, Hirs	M31213.1	5979
211430_s_at	immunoglobulin heavy constant gamma 3 (G3m marker)	M87789.1	3502
211483_x_at	calcium/calmodulin-dependent protein kinase (CaM kinase) II beta	AF081924.1	816
211509_s_at	reticulon 4	AB015639.1	57142
211528_x_at	HLA-G histocompatibility antigen, class I, G	M90685.1	3135
211529_x_at	HLA-G histocompatibility antigen, class I, G	M90684.1	3135
211555_s_at	guanylate cyclase 1, soluble, beta 3	AF020340.1	2983
211564_s_at	LIM domain protein	BC003096.1	8572
211666_x_at	gb:L22453.1 /DEF=Homo sapiens HIV-1 TAR RNA binding protein (TARBP-b) mRNA, co	L22453.1	
211696_x_at	hemoglobin, beta	AF349114.1	3043
211698_at	CREBBP/EP300 inhibitory protein 1	AF349444.1	23741
211699_x_at	hemoglobin, alpha 1	AF349571.1	3039
211719_x_at	fibronectin 1	BC005858.1	2335
211737_x_at	pleiotrophin (heparin binding growth factor 8, neurite growth-promoting factor 1)	BC005916.1	5764
211745_x_at	hemoglobin, alpha 2	BC005931.1	3040
211799_x_at	H.sapiens mRNA for HLA-C alpha chain (Cw*1701)	U62824.1	
211813_x_at	decorin	AF138303.1	1634
211911_x_at	gb:L07950.1 /DEF=Homo sapiens MHC class I HLA B71 mRNA, complete cds. /FEA=CD	L07950.1	
211936_at	heat shock 70kDa protein 5 (glucose-regulated protein, 78kDa)	AF216292.1	3309
211941_s_at	prostatic binding protein	BF686267	5037
211962_s_at	zinc finger protein 36, C3H type-like 1	X79067.1	677
211980_at	collagen, type IV, alpha 1	NM_001845.1	1282
211984_at	Homo sapiens calmodulin-I (CALM1) mRNA, 3'UTR, partial sequence	AI653730	
211985_s_at	Homo sapiens calmodulin-I (CALM1) mRNA, 3'UTR, partial sequence	AI653730	
211991_s_at	major histocompatibility complex, class II, DP alpha 1	M27487.1	3113
211995_x_at	actin, gamma 1	AL567820	71
212063_at	CD44 antigen (homing function and Indian blood group system)	BE903880	960
212085_at	solute carrier family 25 (mitochondrial carrier; adenine nucleotide translocator), member 6	AA916851	293
212094_at	paternally expressed 10	BE858180	23089
212097_at	caveolin 1, caveolae protein, 22kDa	AU147399	857
212131_at	DKFZP434D1335 protein	AL117499.1	26065
212158_at	syndecan 2 (heparan sulfate proteoglycan 1, cell surface-associated, fibroglycan)	J04621.1	6383
212190_at	serine (or cysteine) proteinase inhibitor, clade E (nexin, plasminogen activator inhibitor ty	AL541302	5270
212224_at	aldehyde dehydrogenase 1 family, member A1	NM_000689.1	216
212230_at	phosphatidic acid phosphatase type 2B	AL576654	8613
212298_at	neuropilin 1	BE620457	8829
212464_s_at	fibronectin 1	X02761.1	2335
212560_at	Homo sapiens cDNA: FLJ21930 fis, clone HEP04301, highly similar to HSU90916 Humai	AV728268	
212667_at	myosin IF	AL575922	4542
212732_at	maternally expressed 3	AI950273	55384
212805_at	KIAA0367 protein	AB002365.1	23273
212884_x_at	apolipoprotein E	AI358867	348
212915_at	KIAA1095 protein	AL569804	23024
212977_at	G protein-coupled receptor	AI817041	57007
212992_at	hypothetical protein BC011859	AI935123	113146
213016_at	Homo sapiens clone 24630 mRNA sequence	BF448315	
213135_at	Human clone 23612 mRNA sequence	U90902.1	
213182_x_at	cyclin-dependent kinase inhibitor 1C (p57, Kip2)	R78668	1028
213183_s_at	cyclin-dependent kinase inhibitor 1C (p57, Kip2)	R78668	1028

213187_x_at	ferritin, light polypeptide	BG538564	2512
213241_at	Homo sapiens clone 23785 mRNA sequence	AF035307.1	
213280_at	KIAA1039 protein	AK000478.1	23108
213293_s_at	tripartite motif-containing 22	AA083478	10346
213404_s_at	Ras homolog enriched in brain 2	BF033683	6009
213453_x_at	glyceraldehyde-3-phosphate dehydrogenase	BF689355	2597
213479_at	neuronal pentraxin II	U26662.1	4885
213502_x_at	Homo sapiens cDNA FLJ32313 fis, clone PROST2003232, weakly similar to BETA-GLU(	X03529	
213524_s_at	putative lymphocyte G0/G1 switch gene	NM_015714.1	50486
213553_x_at	apolipoprotein C-I	W79394	341
213655_at	tyrosine 3-monooxygenase/tryptophan 5-monooxygenase activation protein, epsilon poly|	AA502643	7531
213664_at	solute carrier family 1 (neuronal/epithelial high affinity glutamate transporter, system Xag)	AW235061	6505
213701_at	Homo sapiens cDNA FLJ38158 fis, clone DFNES2001091	AW299245	
213757_at	eukaryotic translation initiation factor 5A	BF541557	1984
213791_at	proenkephalin	NM_006211.1	5179
213793_s_at	Homer, neuronal immediate early gene, 1B	BE550452	9456
213841_at	Homo sapiens mRNA, chromosome 1 specific transcript KIAA0510	BE223030	
213847_at	peripherin	NM_006262.1	5630
213869_x_at	Thy-1 cell surface antigen	AA218868	7070
213921_at	somatostatin	NM_001048.1	6750
213943_at	twist homolog (acrocephalosyndactyly 3; Saethre-Chotzen syndrome) (Drosophila)	X99268.1	7291
213975_s_at	tudor repeat associator with PCTAIRE 2	AV711904	23424
214079_at	Homo sapiens cDNA FLJ20338 fis, clone HEP12179	AK000345.1	
214157_at	GNAS complex locus	AA401492	2778
214167_s_at	ribosomal protein, large, P0	AA555113	6175
214218_s_at	Homo sapiens cDNA FLJ30298 fis, clone BRACE2003172	AV699347	
214247_s_at	dickkopf homolog 3 (Xenopus laevis)	AU148057	27122
214315_x_at	calreticulin	AI348935	811
214321_at	nephroblastoma overexpressed gene	BF440025	4856
214414_x_at	hemoglobin, alpha 2	T50399	3040
214428_x_at	complement component 4A	K02403.1	720
214432_at	hypothetical protein MGC13276	NM_000703.1	95633
214451_at	transcription factor AP-2 beta (activating enhancer binding protein 2 beta)	NM_003221.1	7021
214577_at	microtubule-associated protein 1B	BG164365	4131
214607_at	p21 (CDKN1A)-activated kinase 3	AW085556	5063
214610_at	Homo sapiens cDNA FLJ36771 fis, clone ADRGL1000147, moderately similar to CYTOC	AV702430	
214611_at	glutamate receptor, ionotropic, kainate 1	U16125.1	2897
214620_x_at	peptidylglycine alpha-amidating monooxygenase	BF038548	5066
214669_x_at	Homo sapiens isolate donor N clone N168K immunoglobulin kappa light chain variable re	BG485135	
214677_x_at	immunoglobulin lambda joining 3	X57812.1	28831
214678_x_at	zinc finger protein, X-linked	R51161	7543
214723_x_at	KIAA1641 protein	AB046861.1	57730
214761_at	OLF-1/EBF associated zinc finger gene	AW149417	23090
214774_x_at	trinucleotide repeat containing 9	AK027006.1	27324
214836_x_at	immunoglobulin kappa constant	BG536224	3514
214850_at	SMA3	X75940.1	10571
214858_at	Homo sapiens clone 24566 mRNA sequence	AF070536.1	
215049_x_at	CD163 antigen	Z22969.1	9332
215076_s_at	collagen, type III, alpha 1 (Ehlers-Danlos syndrome type IV, autosomal dominant)	AU144167	1281
215108_x_at	trinucleotide repeat containing 9	U80736.1	27324
215121_x_at	immunoglobulin lambda locus	AA680302	3535
215193_x_at	major histocompatibility complex, class II, DR beta 3	AJ297586.1	3125

215311_at	Homo sapiens mRNA full length insert cDNA clone EUROIMAGE 21920	AL109696.1	
215388_s_at	H factor (complement)-like 2	X56210.1	3079
215440_s_at	hypothetical protein FLJ10097	AL523320	56271
215643_at	Homo sapiens cDNA FLJ11740 fis, clone HEMBA1005500	AU145680	
215715_at	solute carrier family 6 (neurotransmitter transporter, noradrenalin), member 2	BC000563.1	6530
215771_x_at	ret proto-oncogene (multiple endocrine neoplasia and medullary thyroid carcinoma 1, Hirs	X15786.1	5979
215946_x_at	Homo sapiens cDNA FLJ32313 fis, clone PROST2003232, weakly similar to BETA-GLU(	AL022324	
215952_s_at	Homo sapiens clone IMAGE 172979	AF090094.1	
216216_at	slit homolog 3 (Drosophila)	AL122074.1	6586
216231_s_at	beta-2-microglobulin	AW188940	567
216248_s_at	nuclear receptor subfamily 4, group A, member 2	S77154.1	4929
216379_x_at	KIAA1919 protein	AK000168.1	91749
216442_x_at	fibronectin 1	AK026737.1	2335
216470_x_at	Consensus includes gb:AF009664 /DEF=Homo sapiens T cell receptor beta locus, 3 tryp	AF009664	
216520_s_at	Consensus includes gb:AF072098 /DEF=Homo sapiens HDCMB21P gene, complete cds	AF072098	
216570_x_at	Consensus includes gb:AL096829 /DEF=Human DNA sequence from clone RP4-595K12	AL096829	
216594_x_at	aldo-keto reductase family 1, member C2 (dihydrodiol dehydrogenase 2; bile acid binding	S68290.1	1646
216598_s_at	chemokine (C-C motif) ligand 2	S69738.1	6347
216623_x_at	trinucleotide repeat containing 9	AK025084.1	27324
216834_at	regulator of G-protein signalling 1	S59049.1	5996
216894_x_at	Consensus includes gb:D64137 /DEF=Human KIP2 gene for Cdk-inhibitor p57KIP2, com	D64137	
216938_x_at	dopamine receptor D2	S69899.1	1813
217022_s_at	Homo sapiens SNC73 protein (SNC73) mRNA, complete cds	S55735.1	
217028_at	Consensus includes gb:AJ224869 /DEF=Homo sapiens CXCR4 gene encoding receptor	AJ224869	
217057_s_at	Consensus includes gb:AF107846 /DEF=Homo sapiens neuroendocrine-specific Golgi pr	AF107846	
217200_x_at	cytochrome b-561	U06715.1	1534
217232_x_at	Consensus includes gb:AF059180 /DEF=Homo sapiens mutant beta-globin (HBB) gene,	AF059180	
217356_s_at	Phosphoglycerate kinase {alternatively spliced} [human, phosphoglycerate kinase deficie	S81916.1	
217414_x_at	Consensus includes gb:V00489 /DEF=Human alpha-globin gene with flanks /FEA=mRNA	V00489	
217466_x_at	Consensus includes gb:L48784 /DEF=050 Homo sapiens cDNA /FEA=mRNA /DB_XREF	L48784	
217621_at	peter pan homolog (Drosophila)	AI399889	56342
217732_s_at	integral membrane protein 2B	AF092128.1	9445
217747_s_at	ribosomal protein S9	NM_001013.1	6203
217767_at	complement component 3	NM_000064.1	718
217771_at	golgi phosphoprotein 2	NM_016548.1	51280
217788_s_at	UDP-N-acetyl-alpha-D-galactosamine:polypeptide N-acetylgalactosaminyltransferase 2 ((	NM_004481.2	2590
217871_s_at	macrophage migration inhibitory factor (glycosylation-inhibiting factor)	NM_002415.1	4282
217897_at	FXYD domain containing ion transport regulator 6	NM_022003.1	53826
217911_s_at	BCL2-associated athanogene 3	NM_004281.1	9531
217957_at	transcription factor IIB	NM_013242.1	29105
217983_s_at	ribonuclease 6 precursor	NM_003730.2	8635
218041_x_at	hypothetical protein PRO1068	NM_018573.1	55439
218147_s_at	glycosyltransferase AD-017	NM_018446.1	55830
218172_s_at	hypothetical protein PRO2577	NM_018630.1	55493
218179_s_at	hypothetical protein FLJ12716	NM_021942.1	60684
218380_at	hypothetical protein PP1044	NM_021730.1	60368
218484_at	NADH:ubiquinone oxidoreductase MLRQ subunit homolog	NM_020142.1	56901
218541_s_at	chromosome 8 open reading frame 4	NM_020130.1	56892
218872_at	hypothetical protein FLJ20607	NM_017899.1	54997
218880_at	FOS-like antigen 2	N36408	2355
218952_at	proprotein convertase subtilisin/kexin type 1 inhibitor	NM_013271.1	27344
218974_at	hypothetical protein FLJ10159	NM_018013.1	55084

219049_at	chondroitin beta1,4 N-acetylgalactosaminyltransferase	NM_018371.1	55790
219117_s_at	FK506 binding protein 11, 19 kDa	NM_016594.1	51303
219196_at	secretogranin III	NM_013243.1	29106
219228_at	zinc finger protein 331; zinc finger protein 463	NM_018555.2	55422
219304_s_at	spinal cord-derived growth factor-B	NM_025208.1	80310
219410_at	hypothetical protein FLJ10134	NM_018004.1	55076
219478_at	WAP four-disulfide core domain 1	NM_021197.1	58189
219534_x_at	cyclin-dependent kinase inhibitor 1C (p57, Kip2)	NM_000076.1	1028
219659_at	ATPase, aminophospholipid transporter-like, Class I, type 8A, member 2	AU146927	51761
219737_s_at	protocadherin 9	AI524125	5101
219738_s_at	protocadherin 9	NM_020403.1	5101
219777_at	hypothetical protein FLJ22690	NM_024711.1	79765
219791_s_at	hypothetical protein FLJ11539	NM_024748.1	79804
219802_at	hypothetical protein FLJ22028	NM_024854.1	79912
219895_at	hypothetical protein FLJ20716	NM_017938.1	55026
219922_s_at	latent transforming growth factor beta binding protein 3	NM_021070.1	4054
220136_s_at	crystallin, beta A2	NM_005209.1	1412
220231_at	G-substrate	NM_006658.1	10842
220370_s_at	KIAA1453 protein	NM_025090.1	57602
220576_at	hypothetical protein FLJ12377	NM_024989.1	80055
220795_s_at	KIAA1446 protein	NM_020836.1	57596
220940_at	KIAA1641 protein	NM_025190.1	57730
220954_s_at	paired immunoglobulin-like receptor beta	NM_013440.1	29990
221127_s_at	regulated in glioma	NM_006394.1	10530
221452_s_at	hypothetical protein MGC1223	NM_030969.1	81853
221476_s_at	ribosomal protein L15	AF279903.1	6138
221651_x_at	immunoglobulin kappa constant	BC005332.1	3514
221671_x_at	immunoglobulin kappa constant	M63438.1	3514
221691_x_at	nucleophosmin (nucleolar phosphoprotein B23, numatrin)	AB042278.1	4869
221701_s_at	hypothetical protein FLJ12541 similar to Stra6	AF352728.1	64220
221728_x_at	Homo sapiens cDNA FLJ30298 fis, clone BRACE2003172	AK025198.1	
221766_s_at	chromosome 6 open reading frame 37	AL078599	55603
221771_s_at	M-phase phosphoprotein, mpp8	BC003542.1	54737
221801_x_at	Homo sapiens cDNA FLJ37371 fis, clone BRAMY2024711, moderately similar to Mus mu	NM_006158.1	
221805_at	neurofilament, light polypeptide 68kDa	NM_006158.1	4747
221916_at	Homo sapiens cDNA FLJ37371 fis, clone BRAMY2024711, moderately similar to Mus mu	BF055311	
222033_s_at	fms-related tyrosine kinase 1 (vascular endothelial growth factor/vascular permeability fac	AA058828	2321
222108_at	Homo sapiens cDNA FLJ35792 fis, clone TESTI2005759	AC004010	
222162_s_at	a disintegrin-like and metalloprotease (reprolysin type) with thrombospondin type 1 motif,	AK023795.1	9510
266_s_at	CD24 antigen (small cell lung carcinoma cluster 4 antigen)	L33930	934
32088_at	basic leucine zipper nuclear factor 1 (JEM-1)	U79751	8548
33494_at	electron-transferring-flavoprotein dehydrogenase	S69232	2110
34031_i_at	cerebral cavernous malformations 1	U90268	889
38241_at	butyrophilin, subfamily 3, member A3	U90548	10384
39248_at	aquaporin 3	N74607	360
41386_i_at	KIAA0346 protein	AB002344	23135
