# Supplementary material for: A HIF1α Regulatory Loop Links Hypoxia and Mitochondrial Signals in Pheochromocytomas
Source: PLoS Genet. 2005 Jul 25;1(1):e8. doi: 10.1371/journal.pgen.0010008 (PMC1183527; doi:10.1371/journal.pgen.0010008)
Supplement: Dataset S3 — (27 KB PDF) [file pgen.0010008.sd003.pdf]

Suppl. Data 3. Validation by real time PCR and Western analyses of genes differentially expressed in pheochromocytomas

A)

Gene	Predominant Cluster by Microarray	Predominant Cluster by RT-PCR	P value
Aquaporin 3	1	1	8.9E-07
Cytochrome b561	2	2	4.1E-05
p57/kip1	2	2	0.01
Slit homolog 3	2	2	0.01

B)

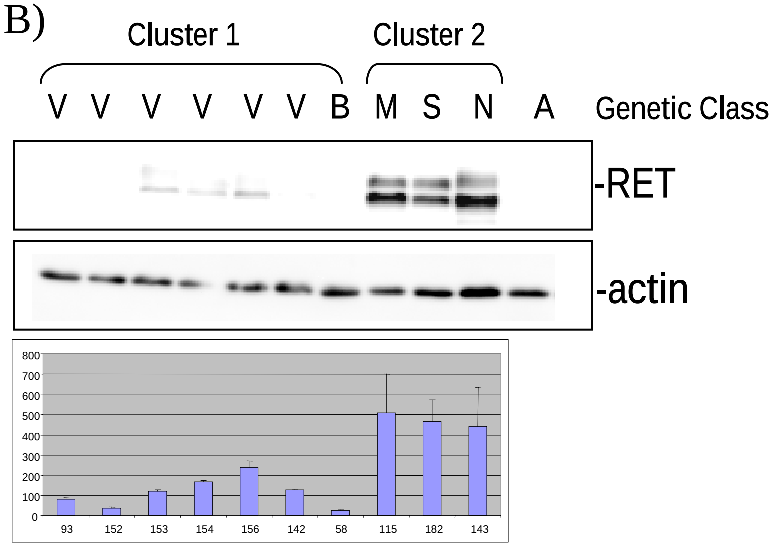

A) Confirmation of microarray expression by real time-PCR of genes with predominant expression in Cluster 1 or Cluster 2 in 20 tumors (10 from Cluster 1 and 10 from Cluster 2) from the pheochromocytoma cohort. **B)** Expression of RET, one of the genes highly expressed in Cluster 2 tumors, in whole cell lysates of a group of tumor samples used for the transcription analysis. RET protein is overexpressed in most tumors from cluster 2. In contrast, RET was undetectable or expressed at low levels in most samples belonging to cluster 1 and normal adrenal medulla used as control. Corresponding expression of the tumors on U133A microarray (averaged results from 3 independent RET probes) is displayed below and shows high correspondence with RET protein expression. (V=VHL, B=SDHB, M=MEN2, N=NF1, S=sporadic, A=normal adrenal medulla; sample ID is indicated at the bottom of each column in the graph).
