# Supplementary material for: A HIF1α Regulatory Loop Links Hypoxia and Mitochondrial Signals in Pheochromocytomas
Source: PLoS Genet. 2005 Jul 25;1(1):e8. doi: 10.1371/journal.pgen.0010008 (PMC1183527; doi:10.1371/journal.pgen.0010008)
Supplement: Dataset S4 — (345 KB PDF) [file pgen.0010008.sd004.pdf]

Genes that discriminate MEN2 vs. VHL tumors by supervised learning method
Marker analysis for the Pheochromocytoma MEN2 vs. VHL (19 samples, with 10 MEN2A and 9 VHL
Data thresholded (10 minimum and 16000 maximum) and filtered (5 fold minimum variation and 50 minimum absolute difference) (14811 fe
Marker analysis using mean based signal-to-noise ratio with significance evaluated with 2000 permutatior

#	Class	Score	Probe Set na	Gene Name
1	MEN2	1.7	205230_at	likely ortholog of mouse rabphilin 3A
2	MEN2	1.58	209160_at	aldo-keto reductase family 1, member C3
3	MEN2	1.4	214247_s_at	dickkopf homolog 3 (Xenopus laevis
4	MEN2	1.36	216594_x_at	aldo-keto reductase family 1, member C2
5	MEN2	1.34	221701_s_at	hypothetical protein FLJ12541 similar to Stra6
6	MEN2	1.26	204151_x_at	aldo-keto reductase family 1, member C1
7	MEN2	1.23	213348_at	cyclin-dependent kinase inhibitor 1C (p57, Kip2
8	MEN2	1.23	219250_s_at	fibronectin leucine rich transmembrane protein 3
9	MEN2	1.2	212905_at	likely ortholog of mouse variant polyadenylation protein CSTF-64
10	MEN2	1.17	214822_at	hypothetical protein from clone 24828
11	MEN2	1.16	219534_x_at	cyclin-dependent kinase inhibitor 1C (p57, Kip2
12	MEN2	1.16	219271_at	hypothetical protein FLJ12691
13	MEN2	1.15	202196_s_at	dickkopf homolog 3 (Xenopus laevis
14	MEN2	1.09	209699_x_at	aldo-keto reductase family 1, member C2
15	MEN2	1.08	215771_x_at	ret proto-oncogene
16	MEN2	1.08	209794_at	SLIT-ROBO Rho GTPase-activating protein 3
17	MEN2	1.07	211653_x_at	aldo-keto reductase family 1, member C2
18	MEN2	1.07	216894_x_at	unknown EST, accession number D64137
19	MEN2	1.06	203440_at	cadherin 2, type 1, N-cadherin (neuronal
20	MEN2	1.04	214609_at	aristaless homeobox (Drosophila)
21	MEN2	1.04	211421_s_at	ret proto-oncogene
22	MEN2	1.03	221127_s_at	regulated in glioma
23	MEN2	1.01	200965_s_at	actin binding LIM protein 1
24	MEN2	1.01	201990_s_at	cAMP responsive element binding protein-like 2
25	MEN2	0.99	210778_s_at	MAX dimerization protein 4
26	MEN2	0.98	206793_at	phenylethanolamine N-methyltransferase
27	MEN2	0.98	219389_at	hypothetical protein FLJ10052
28	MEN2	0.98	205879_x_at	ret proto-oncogene
29	MEN2	0.98	213182_x_at	cyclin-dependent kinase inhibitor 1C (p57, Kip2
30	MEN2	0.98	221974_at	imprinted in Prader-Willi syndrome
31	MEN2	0.97	206159_at	growth differentiation factor 10
32	MEN2	0.96	203335_at	phytanoyl-CoA hydroxylase (Refsum disease
33	MEN2	0.95	218087_s_at	sorbin and SH3 domain containing 1
34	MEN2	0.95	222103_at	activating transcription factor 1
35	MEN2	0.95	212240_s_at	phosphoinositide-3-kinase, regulatory subunit, polypeptide 1 (p85 alpha
36	MEN2	0.92	210078_s_at	potassium voltage-gated channel, shaker-related subfamily, beta member
37	MEN2	0.92	209609_s_at	mitochondrial ribosomal protein L9
38	MEN2	0.91	209286_at	Homo sapiens cDNA FLJ31353 fis, clone MESAN2000264
39	MEN2	0.91	217621_at	peter pan homolog (Drosophila)
40	MEN2	0.91	212686_at	KIAA1157 protein
41	MEN2	0.9	218168_s_at	chaperone, ABC1 activity of bc1 complex like (S. pombe
42	MEN2	0.89	219619_at	Di-Ras2
43	MEN2	0.89	209075_s_at	nitrogen fixation cluster-like
44	MEN2	0.89	206137_at	regulating synaptic membrane exocytosis 2
45	MEN2	0.88	201247_at	Homo sapiens mRNA; cDNA DKFZp434N1610 (from clone DKFZp434N1610
46	MEN2	0.88	204041_at	monoamine oxidase B
47	MEN2	0.88	200748_s_at	ferritin, heavy polypeptide 1
48	MEN2	0.87	210059_s_at	mitogen-activated protein kinase 13
49	MEN2	0.87	201738_at	translation factor sui1 homolog
50	MEN2	0.87	211382_s_at	transforming, acidic coiled-coil containing protein 2
51	MEN2	0.87	204905_s_at	eukaryotic translation elongation factor 1 epsilon 1
52	MEN2	0.86	203519_s_at	regulator of nonsense transcripts 2
53	MEN2	0.86	206634_at	sine oculis homeobox homolog 3 (Drosophila
54	MEN2	0.86	204121_at	growth arrest and DNA-damage-inducible, gamma
55	MEN2	0.85	204148_s_at	POM (POM121 homolog, rat) and ZP3 fusion
56	MEN2	0.85	204362_at	src family associated phosphoprotein 2
57	MEN2	0.85	217772_s_at	mitochondrial carrier homolog 2
58	MEN2	0.84	206730_at	glutamate receptor, ionotrophic, AMPA 3
59	MEN2	0.84	219714_s_at	calcium channel, voltage-dependent, alpha 2/delta 3 subun
60	MEN2	0.84	205580_s_at	histamine receptor H1
61	MEN2	0.83	204170_s_at	CDC28 protein kinase 2
62	MEN2	0.83	208792_s_at	clusterin (complement lysis inhibitor, SP-40,40
63	MEN2	0.83	222043_at	clusterin (complement lysis inhibitor, SP-40,40
64	MEN2	0.82	221190_s_at	colon cancer-associated protein Mic1
65	MEN2	0.82	219933_at	glutaredoxin 2
66	MEN2	0.82	203584_at	KIAA0103 gene product
67	MEN2	0.82	218980_at	hypothetical protein FLJ22297
68	MEN2	0.81	206001_at	neuropeptide Y
69	MEN2	0.81	209869_at	adrenergic, alpha-2A-, receptor
70	MEN2	0.81	202201_at	biliverdin reductase B (flavin reductase (NADPH)
71	MEN2	0.8	214920_at	Homo sapiens cDNA FLJ11022 fis, clone PLACE1003771
72	MEN2	0.8	219289_at	hypothetical protein FLJ20718
73	MEN2	0.8	213637_at	ESTs,accession number BE503392
74	MEN2	0.79	218450_at	heme binding protein 1
75	MEN2	0.79	202289_s_at	transforming, acidic coiled-coil containing protein 2
76	MEN2	0.79	206662_at	glutaredoxin (thioltransferase)
77	MEN2	0.78	202794_at	inositol polyphosphate-1-phosphatase
78	MEN2	0.78	205413_at	chromosome 11 open reading frame 8
79	MEN2	0.78	206478_at	KIAA0125 gene product
80	MEN2	0.78	218518_at	chromosome 5 open reading frame 5
81	MEN2	0.78	209785_s_at	phospholipase A2, group IVC (cytosolic, calcium-independent
82	MEN2	0.78	219310_at	chromosome 20 open reading frame 39
83	MEN2	0.78	202363_at	sparc/osteonectin, cwcv and kazal-like domains proteoglycan (testican
84	MEN2	0.77	218330_s_at	neuron navigator 2
85	MEN2	0.77	221791_s_at	hypothetical protein HSPC016
86	MEN2	0.77	200953_s_at	cyclin D2
87	MEN2	0.77	212736_at	hypothetical gene BC008967
88	MEN2	0.76	200704_at	LPS-induced TNF-alpha factor
89	MEN2	0.76	203628_at	insulin-like growth factor 1 receptor
90	MEN2	0.76	204615_x_at	isopentenyl-diphosphate delta isomerase
91	MEN2	0.76	207922_s_at	macrophage erythroblast attacher
92	MEN2	0.76	221861_at	Homo sapiens mRNA; cDNA DKFZp762M127 (from clone DKFZp762M127
93	MEN2	0.75	209242_at	paternally expressed 3
94	MEN2	0.75	203281_s_at	ubiquitin-activating enzyme E1-like

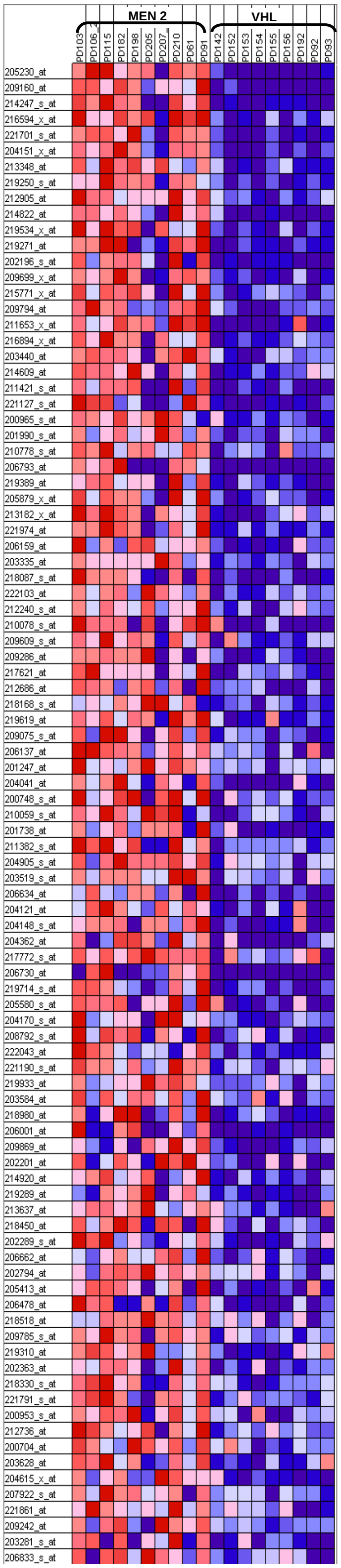

95	MEN2	0.75	206833_s_at	acylphosphatase 2, muscle type
96	MEN2	0.74	217773_s_at	NADH dehydrogenase (ubiquinone) 1 alpha subcomplex, 4, 9kD
97	MEN2	0.74	208881_x_at	isopentenyl-diphosphate delta isomerase
98	MEN2	0.74	204851_s_at	doublecortex; lissencephaly, X-linked (doublecortin
99	MEN2	0.74	215715_at	solute carrier family 6 (neurotransmitter transporter, noradrenalin), member [illegible]
100	MEN2	0.74	201814_at	TBC1 domain family, member 5
101	VHL	2.05	204597_x_at	stanniocalcin 1
102	VHL	1.69	207543_s_at	procollagen-proline, 2-oxoglutarate 4-dioxygenase (proline 4-hydroxylase
103	VHL	1.61	202998_s_at	lysyl oxidase-like 2
104	VHL	1.54	218974_at	hypothetical protein FLJ10159
105	VHL	1.46	201579_at	FAT tumor suppressor homolog 1 (Drosophila
106	VHL	1.44	202934_at	hexokinase 2
107	VHL	1.39	203438_at	stanniocalcin 2
108	VHL	1.27	217419_x_at	agrin
109	VHL	1.26	200941_at	heat shock factor binding protein 1
110	VHL	1.24	214096_s_at	serine hydroxymethyltransferase 2 (mitochondrial
111	VHL	1.24	214903_at	Homo sapiens clone 24527 mRNA sequence
112	VHL	1.22	212285_s_at	agrin
113	VHL	1.16	203006_at	inositol polyphosphate-5-phosphatase, 40kDa
114	VHL	1.14	205656_at	protocadherin 17
115	VHL	1.14	200632_s_at	N-myc downstream regulated gene 1
116	VHL	1.14	38671_at	KIAA0620 protein
117	VHL	1.09	204348_s_at	adenylate kinase 3
118	VHL	1.09	201102_s_at	phosphofructokinase, liver
119	VHL	1.07	214081_at	tumor endothelial marker 7 precursor
120	VHL	1.07	221261_x_at	MAGE-E1 protein
121	VHL	1.07	212047_s_at	DKFZP566H073 protein
122	VHL	1.05	207196_s_at	Nef-associated factor 1
123	VHL	1.04	201917_s_at	hypothetical protein FLJ10618
124	VHL	1.03	221203_s_at	hypothetical protein FLJ10201
125	VHL	1.03	218825_at	NEU1 protein
126	VHL	1.02	220584_at	hypothetical protein FLJ22184
127	VHL	1.02	202192_s_at	growth arrest-specific 7
128	VHL	1.02	205013_s_at	adenosine A2a receptor
129	VHL	1	201918_at	hypothetical protein FLJ10618
130	VHL	1	214803_at	Homo sapiens mRNA; cDNA DKFZp564N1116 (from clone DKFZp564N1116
131	VHL	1	35156_at	ESTs, Highly similar to T08701 hypothetical protein DKFZp564N123.1 - human
132	VHL	1	221830_at	Homo sapiens cDNA FLJ37267 fis, clone BRAMY2011297
133	VHL	0.99	219165_at	PDZ-LIM protein mystique
134	VHL	0.98	205978_at	klotho
135	VHL	0.98	204595_s_at	stanniocalcin 1
136	VHL	0.96	212707_s_at	Ca2+-promoted Ras inactivator
137	VHL	0.91	219700_at	tumor endothelial marker 7 precursor
138	VHL	0.91	200745_s_at	guanine nucleotide binding protein (G protein), beta polypeptide
139	VHL	0.91	204337_at	regulator of G-protein signalling 4
140	VHL	0.91	218916_at	hypothetical protein FLJ23436
141	VHL	0.9	214177_s_at	hematopoietic PBX-interacting protein
142	VHL	0.9	215543_s_at	like-glycosyltransferase
143	VHL	0.89	216268_s_at	jagged 1 (Alagille syndrome)
144	VHL	0.89	221904_at	hypothetical protein MGC21688
145	VHL	0.89	205902_at	potassium intermediate/small conductance calcium-activated channel, subfamily
146	VHL	0.88	212730_at	desmuslin
147	VHL	0.87	219315_s_at	hypothetical protein FLJ20898
148	VHL	0.87	206385_s_at	ankyrin 3, node of Ranvier (ankyrin G
149	VHL	0.87	201867_s_at	transducin (beta)-like 1X-linked
150	VHL	0.87	65718_at	tumor endothelial marker 5 precursor
151	VHL	0.87	218424_s_at	dudulin 2
152	VHL	0.86	218484_at	NADH:ubiquinone oxidoreductase MLRQ subunit homolo
153	VHL	0.86	208674_x_at	dolichyl-diphosphooligosaccharide-protein glycosyltransferas
154	VHL	0.86	204078_at	nucleolar autoantigen (55kD) similar to rat synaptonemal complex protei
155	VHL	0.86	209392_at	ectonucleotide pyrophosphatase/phosphodiesterase 2 (autotaxin
156	VHL	0.86	218975_at	collagen, type V, alpha 3
157	VHL	0.86	200827_at	procollagen-lysine, 2-oxoglutarate 5-dioxygenase (lysine hydroxylase
158	VHL	0.86	204784_s_at	myeloid leukemia factor 1
159	VHL	0.84	210095_s_at	insulin-like growth factor binding protein [illegible]
160	VHL	0.84	212323_s_at	KIAA0453 protein
161	VHL	0.84	208850_s_at	Thy-1 cell surface antigen
162	VHL	0.84	219410_at	hypothetical protein FLJ10134
163	VHL	0.84	214157_at	GNAS complex locus
164	VHL	0.83	213967_at	Homo sapiens clone 24468 mRNA sequence
165	VHL	0.83	203230_at	dishevelled, dsh homolog 1 (Drosophila
166	VHL	0.83	212985_at	Homo sapiens mRNA; cDNA DKFZp434E033 (from clone DKFZp434E033
167	VHL	0.82	52255_s_at	collagen, type V, alpha 3
168	VHL	0.81	215083_at	Homo sapiens mRNA; cDNA DKFZp564F133 (from clone DKFZp564F133
169	VHL	0.81	210776_x_at	transcription factor 3 (E2A immunoglobulin enhancer binding factors E12/E47
170	VHL	0.81	209082_s_at	collagen, type XVIII, alpha 1
171	VHL	0.81	212888_at	Homo sapiens cDNA FLJ33775 fis, clone BRSSN2000498
172	VHL	0.81	208824_x_at	PCTAIRE protein kinase 1
173	VHL	0.81	210512_s_at	vascular endothelial growth factor
174	VHL	0.81	210070_s_at	carnitine palmitoyltransferase I, muscle
175	VHL	0.8	220975_s_at	C1q and tumor necrosis factor related protein 1
176	VHL	0.8	205031_at	ephrin-B3
177	VHL	0.8	210740_s_at	inositol 1,3,4-triphosphate 5/6 kinase
178	VHL	0.8	205286_at	transcription factor AP-2 gamma (activating enhancer binding protein 2 gamma
179	VHL	0.79	213355_at	alpha2,3-sialyltransferase
180	VHL	0.79	218001_at	mitochondrial ribosomal protein S2
181	VHL	0.79	215045_at	trinucleotide repeat containing 4
182	VHL	0.79	214098_at	KIAA1107 protein
183	VHL	0.79	220974_x_at	similar to rat tricarboxylate carrier-like protein
184	VHL	0.79	204301_at	KIAA0711 gene product
185	VHL	0.78	209651_at	transforming growth factor beta 1 induced transcript 1
186	VHL	0.78	213869_x_at	Thy-1 cell surface antigen
187	VHL	0.78	214468_at	myosin, heavy polypeptide 6, cardiac muscle, alpha
188	VHL	0.78	213912_at	KIAA0984 protein
189	VHL	0.78	219116_s_at	hypothetical protein FLJ10704
190	VHL	0.78	203673_at	thyroglobulin
191	VHL	0.77	201449_at	Homo sapiens cDNA FLJ36425 fis, clone THYMU2011482
192	VHL	0.77	212058_at	KIAA0332 protein
193	VHL	0.77	210150_s_at	laminin, alpha 5
194	VHL	0.76	221814_at	tumor endothelial marker 5 precursor
195	VHL	0.76	205224_at	surfeit 2
196	VHL	0.76	200088_x_at	ribosomal protein L12
197	VHL	0.76	202593_s_at	membrane interacting protein of RGS16
198	VHL	0.76	206172_at	interleukin 13 receptor, alpha 2
199	VHL	0.76	221427_s_at	likely ortholog of mouse Paneth cell enhanced expression
200	VHL	0.76	205131_x_at	stem cell growth factor; lymphocyte secreted C-type lectin

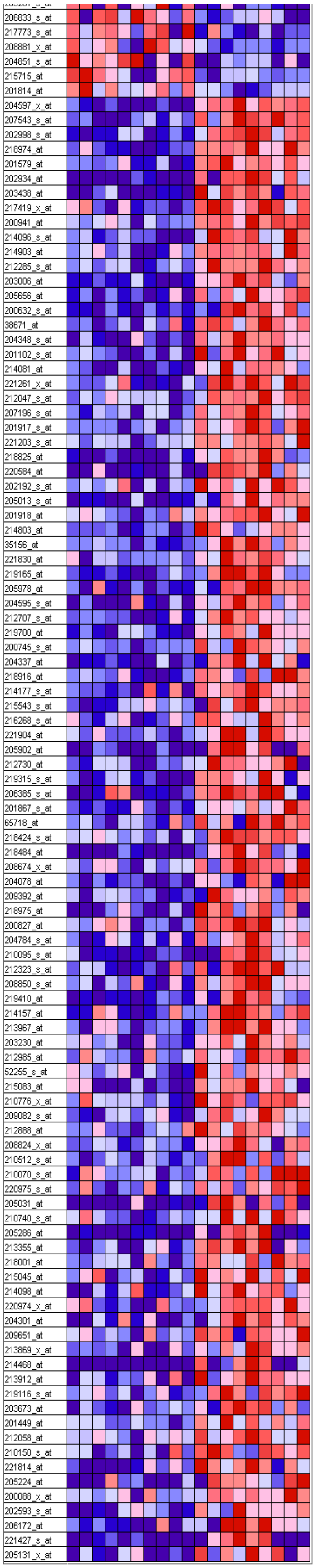

Genes that discriminate MEN2 vs. SDH tumors by supervised learning method
Marker analysis for the Pheochromocytoma MEN2 vs. SDH (16 samples with 10 MEN2A, 5 SDHB, and 1 SDHD
Data thresholded (10 minimum and 16000 maximum) and filtered (5 fold minimum variation and 50 minimum absolute difference) (13153 features passed filterin
Marker analysis using mean based signal-to-noise ratio with significance evaluated with 2000 permutation

#	Class	Score	Feature	Gene Name
1	MEN2	1.7	210059_s_a	mitogen-activated protein kinase 13
2	MEN2	1.65	205230_at	likely ortholog of mouse rabphilin 3A
3	MEN2	1.63	216594_x_a	aldo-keto reductase family 1, member C2
4	MEN2	1.63	213348_at	cyclin-dependent kinase inhibitor 1C (p57, Kip2
5	MEN2	1.6	204121_at	growth arrest and DNA-damage-inducible, gamma
6	MEN2	1.47	220136_s_a	crystallin, beta A2
7	MEN2	1.46	206137_at	regulating synaptic membrane exocytosis 2
8	MEN2	1.44	209160_at	aldo-keto reductase family 1, member C3
9	MEN2	1.44	213182_x_a	cyclin-dependent kinase inhibitor 1C (p57, Kip2
10	MEN2	1.44	210253_at	HIV-1 Tat interactive protein 2, 30kDa
11	MEN2	1.43	218985_at	solute carrier family 2, (facilitated glucose transporter) member 8
12	MEN2	1.4	219534_x_a	cyclin-dependent kinase inhibitor 1C (p57, Kip2
13	MEN2	1.38	220340_at	hypothetical protein FLJ13687
14	MEN2	1.37	201430_s_a	dihydropyrimidinase-like 3
15	MEN2	1.36	215299_x_a	sulfotransferase family, cytosolic, 1A, phenol-preferring, member 1
16	MEN2	1.34	203423_at	retinol binding protein 1, cellular
17	MEN2	1.34	221701_s_a	hypothetical protein FLJ12541 similar to Stra6
18	MEN2	1.33	219619_at	Di-Ras2
19	MEN2	1.32	214822_at	hypothetical protein from clone 24828
20	MEN2	1.31	215771_x_a	ret proto-oncogene
21	MEN2	1.31	206159_at	growth differentiation factor 10
22	MEN2	1.28	216894_x_a	accession number D64137
23	MEN2	1.28	207468_s_a	secreted frizzled-related protein 5
24	MEN2	1.27	206849_at	gamma-aminobutyric acid (GABA) A receptor, gamma 2
25	MEN2	1.26	205879_x_a	ret proto-oncogene
26	MEN2	1.24	218251_at	hypothetical protein STRAIT11499
27	MEN2	1.24	221226_s_a	putative acid-sensing ion channel
28	MEN2	1.23	219821_s_a	hypothetical protein FLJ20330
29	MEN2	1.23	212701_at	talin 2
30	MEN2	1.22	219250_s_a	fibronectin leucine rich transmembrane protein 3
31	MEN2	1.2	205579_at	histamine receptor H1
32	MEN2	1.2	202908_at	Wolfram syndrome 1 (wolframin)
33	MEN2	1.19	200965_s_a	actin binding LIM protein 1
34	MEN2	1.19	209883_at	chromosome 1 open reading frame 17
35	MEN2	1.17	219222_at	ribokinase
36	MEN2	1.17	214046_at	Homo sapiens mRNA; cDNA DKFZp686F1844 (from clone DKFZp686F1844
37	MEN2	1.16	209448_at	HIV-1 Tat interactive protein 2, 30kDa
38	MEN2	1.16	207770_x_a	chorionic somatomammotropin hormone 2
39	MEN2	1.15	202308_at	sterol regulatory element binding transcription factor 1
40	MEN2	1.15	207981_s_a	estrogen-related receptor gamma
41	MEN2	1.14	202597_at	ESTs
42	MEN2	1.13	210058_at	mitogen-activated protein kinase 13
43	MEN2	1.12	203010_at	signal transducer and activator of transcription 5A
44	MEN2	1.12	211421_s_a	ret proto-oncogene
45	MEN2	1.12	49679_s_at	matrix metalloproteinase 24 (membrane-inserted)
46	MEN2	1.11	219806_s_a	FN5 protein
47	MEN2	1.11	205775_at	DNA segment on chromosome 6(unique) 2654 expressed sequenc
48	MEN2	1.11	204400_at	signal transduction protein (SH3 containing
49	MEN2	1.1	207260_at	FEV protein
50	MEN2	1.08	204041_at	monoamine oxidase B
51	MEN2	1.08	213183_s_a	cyclin-dependent kinase inhibitor 1C (p57, Kip2
52	MEN2	1.07	202718_at	insulin-like growth factor binding protein 2, 36kDa
53	MEN2	1.07	218545_at	hypothetical protein FLJ11088
54	MEN2	1.07	216938_x_a	dopamine receptor D2
55	MEN2	1.06	206478_at	KIAA0125 gene product
56	MEN2	1.06	217118_s_a	KIAA0930 protein
57	MEN2	1.06	219143_s_a	hypothetical protein FLJ20374
58	MEN2	1.06	219463_at	chromosome 20 open reading frame 103
59	MEN2	1.05	207180_s_a	HIV-1 Tat interactive protein 2, 30kDa
60	MEN2	1.05	215411_s_a	chromosome 6 open reading frame 4
61	MEN2	1.04	220227_at	hypothetical protein FLJ22202
62	MEN2	1.04	208404_x_a	potassium inwardly-rectifying channel, subfamily J, member 5
63	MEN2	1.04	207240_s_a	luteinizing hormone/choriogonadotropin recepto
64	MEN2	1.03	218552_at	hypothetical protein FLJ10948
65	MEN2	1.03	206793_at	phenylethanolamine N-methyltransferase
66	MEN2	1.03	205766_at	titin-cap (telethonin)
67	MEN2	1.01	212240_s_a	phosphoinositide-3-kinase, regulatory subunit, polypeptide 1 (p85 alpha
68	MEN2	1.01	204067_at	sulfite oxidase
69	MEN2	1.01	213307_at	SH3 and multiple ankyrin repeat domains 2
70	MEN2	1.01	222043_at	clusterin (complement lysis inhibitor, SP-40,40, sulfated glycoprotein 2
71	MEN2	1	205518_s_a	cytidine monophosphate-N-acetylneuraminic acid hydroxylase
72	MEN2	1	208121_s_a	accession number NM_002848.2
73	MEN2	0.99	219572_at	hypothetical protein FLJ20761
74	MEN2	0.98	205575_at	C1q-related factor
75	MEN2	0.98	204151_x_a	aldo-keto reductase family 1, member C1
76	MEN2	0.98	218168_s_a	chaperone, ABC1 activity of bc1 complex like (S. pombe
77	MEN2	0.97	211653_x_a	aldo-keto reductase family 1, member C2
78	MEN2	0.97	201990_s_a	cAMP responsive element binding protein-like 2
79	MEN2	0.97	221861_at	Homo sapiens mRNA; cDNA DKFZp762M127 (from clone DKFZp762M127
80	MEN2	0.97	219310_at	chromosome 20 open reading frame 39
81	MEN2	0.97	219945_at	DEAD/H (Asp-Glu-Ala-Asp/His) box polypeptide 25
82	MEN2	0.96	213572_s_a	serine (or cysteine) proteinase inhibitor, clade B (ovalbumin), member
83	MEN2	0.96	203962_s_a	nebulette
84	MEN2	0.96	209699_x_a	aldo-keto reductase family 1, member C2
85	MEN2	0.96	206189_at	unc-5 homolog B (C. elegans)
86	MEN2	0.96	205051_s_a	v-kit Hardy-Zuckerman 4 feline sarcoma viral oncogene homolog
87	MEN2	0.96	219714_s_a	calcium channel, voltage-dependent, alpha 2/delta 3 subun
88	MEN2	0.96	207426_s_a	tumor necrosis factor (ligand) superfamily, member 4
89	MEN2	0.95	205589_at	myosin, light polypeptide 3, alkali; ventricular, skeletal, slow
90	MEN2	0.95	58916_at	hypothetical protein MGC2376
91	MEN2	0.95	215056_at	Human clone 23695 mRNA sequence
92	MEN2	0.94	219505_at	cat eye syndrome chromosome region, candidate 1
93	MEN2	0.94	216376_x_a	accession number AC006371
94	MEN2	0.94	206433_s_a	testican 3
95	MEN2	0.94	57540_at	ribokinase
96	MEN2	0.94	207725_at	POU domain, class 4, transcription factor 2
97	MEN2	0.94	206547_s_a	protein phosphatase, EF hand calcium-binding domain
98	MEN2	0.93	210579_s_a	tripartite motif-containing 10

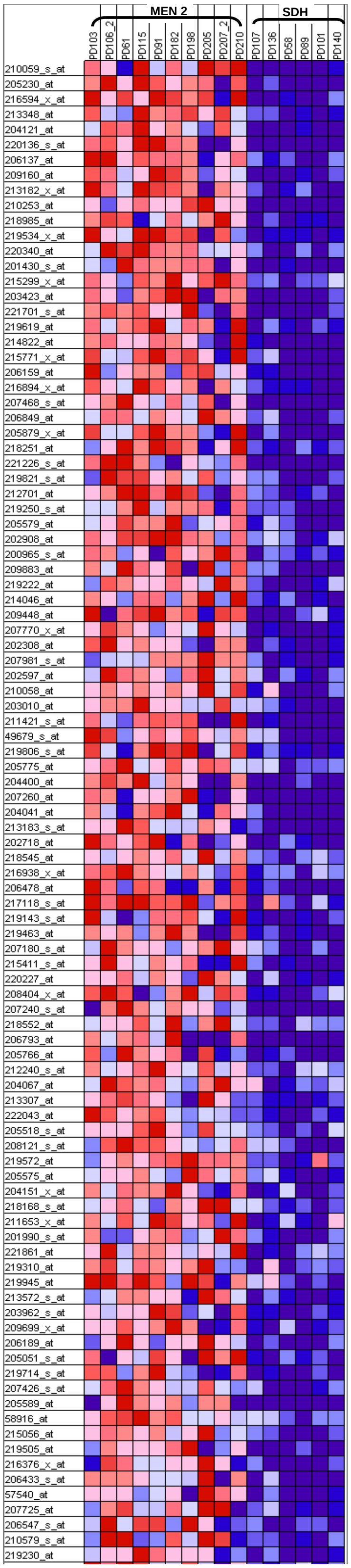

99	MEN2	0.93	219230_at	hypothetical protein FLJ10970
100	MEN2	0.93	220462_at	hypothetical protein FLJ11703
101	MEN2	0.93	207110_at	potassium inwardly-rectifying channel, subfamily J, member 1
102	MEN2	0.93	220153_at	lysosomal apyrase-like protein 1
103	MEN2	0.93	209035_at	midkine (neurite growth-promoting factor 2
104	MEN2	0.93	214247_s_a	dickkopf homolog 3 (Xenopus laevis
105	MEN2	0.92	216259_at	Human clone IMAGE:35527 unknown protein mRNA, partial cd
106	MEN2	0.92	215028_at	sema domain, transmembrane domain (TM), and cytoplasmic domain, (semaphorin) 6A
107	MEN2	0.91	214608_s_a	eyes absent homolog 1 (Drosophila)
108	MEN2	0.91	206051_at	ELAV (embryonic lethal, abnormal vision, Drosophila)-like 4 (Hu antigen D
109	MEN2	0.91	204142_at	rTS beta protein
110	MEN2	0.91	202196_s_a	dickkopf homolog 3 (Xenopus laevis
111	MEN2	0.91	217606_at	Homo sapiens mRNA; cDNA DKFZp686P24158 (from clone DKFZp686P24158
112	MEN2	0.9	202366_at	acyl-Coenzyme A dehydrogenase, C-2 to C-3 short chain
113	MEN2	0.9	203671_at	thiopurine S-methyltransferase
114	MEN2	0.9	214582_at	phosphodiesterase 3B, cGMP-inhibited
115	MEN2	0.9	213197_at	astrotactin
116	MEN2	0.9	208426_x_a	killer cell immunoglobulin-like receptor, two domains, long cytoplasmic tail, 4
117	MEN2	0.9	217561_at	ESTs, Highly similar to 1005250A calcitonin gene related peptide
118	MEN2	0.9	214529_at	thyroid stimulating hormone, beta
119	MEN2	0.9	214322_at	calcium/calmodulin-dependent protein kinase (CaM kinase) II gamm
120	MEN2	0.89	221328_at	claudin 17
121	MEN2	0.89	215174_at	ESTs, Weakly similar to FMO3_HUMAN Dimethylaniline monooxygenase
122	MEN2	0.89	208065_at	sialyltransferase 8C (alpha2,3Galbeta1,4GlcNAcalpha 2,8-sialyltransferase
123	MEN2	0.88	205150_s_a	KIAA0644 gene product
124	MEN2	0.88	213963_s_a	sin3-associated polypeptide, 30kDa
125	MEN2	0.88	221500_s_a	ESTs, Moderately similar to hypothetical protein FLJ20489 [Homo sapiens
126	MEN2	0.88	202974_at	membrane protein, palmitoylated 1, 55kDa
127	MEN2	0.88	207382_at	tumor protein p63
128	MEN2	0.88	206119_at	betaine-homocysteine methyltransferase
129	MEN2	0.88	204417_at	galactosylceramidase (Krabbe disease)
130	MEN2	0.88	205481_at	adenosine A1 receptor
131	MEN2	0.88	215496_at	KIAA1053 protein
132	MEN2	0.87	213448_at	Homo sapiens cDNA FLJ31688 fis, clone NT2RI200552(
133	MEN2	0.87	202321_at	geranylgeranyl diphosphate synthase 1
134	MEN2	0.87	214920_at	Homo sapiens cDNA FLJ11022 fis, clone PLACE100377
135	MEN2	0.87	204675_at	steroid-5-alpha-reductase, alpha polypeptide 1
136	MEN2	0.87	216614_at	Homo sapiens mRNA; cDNA DKFZp564F212 (from clone DKFZp564F212
137	MEN2	0.87	203911_at	RAP1, GTPase activating protein 1
138	MEN2	0.87	205117_at	fibroblast growth factor 1 (acidic)
139	MEN2	0.87	220751_s_a	chromosome 5 open reading frame 4
140	MEN2	0.86	209966_x_a	estrogen-related receptor gamma
141	MEN2	0.86	215856_at	Homo sapiens cDNA FLJ38113 fis, clone D3OST200241
142	MEN2	0.86	207221_at	coagulation factor II (thrombin) receptor-like 3
143	MEN2	0.86	210078_s_a	potassium voltage-gated channel, shaker-related subfamily, beta member
144	MEN2	0.86	213479_at	neuronal pentraxin I
145	MEN2	0.86	217501_at	WD40 protein Ciao1
146	MEN2	0.86	218900_at	cyclin M4
147	MEN2	0.85	206590_x_a	dopamine receptor D2
148	MEN2	0.85	211184_s_a	PDZ-73 protein
149	MEN2	0.85	221394_at	G protein-coupled receptor 58
150	MEN2	0.85	216467_s_a	Homo sapiens mRNA; cDNA DKFZp564L102 (from clone DKFZp564L102
151	MEN2	0.85	208369_s_a	glutaryl-Coenzyme A dehydrogenase
152	MEN2	0.85	221953_s_a	matrix metalloproteinase 24 (membrane-inserted)
153	MEN2	0.85	47069_at	Rho GTPase activating protein 8
154	MEN2	0.85	205264_at	CD3-epsilon-associated protein; antisense to ERCC-1
155	MEN2	0.85	221209_s_a	otoraplin
156	MEN2	0.85	220739_s_a	cyclin M3
157	MEN2	0.85	208227_x_a	a disintegrin and metalloproteinase domain 22
158	MEN2	0.85	203168_at	cAMP responsive element binding protein-like 1
159	MEN2	0.85	206730_at	glutamate receptor, ionotrophic, AMPA 3
160	MEN2	0.84	217124_at	hypothetical protein DKFZp434P1219
161	MEN2	0.84	213894_at	KIAA0960 protein
162	MEN2	0.84	214735_at	phosphoinositide-binding protein PIP3-E
163	MEN2	0.84	200884_at	creatine kinase, brain
164	MEN2	0.84	210311_at	fibroblast growth factor 5
165	MEN2	0.84	219561_at	COPZ2 for nonclathrin coat protein zeta-COP
166	MEN2	0.84	214643_x_a	bridging integrator 1
167	MEN2	0.83	205471_s_a	dachshund homolog (Drosophila
168	MEN2	0.83	215715_at	solute carrier family 6 (neurotransmitter transporter, noradrenalin), member 2
169	MEN2	0.83	211975_at	zinc finger protein 289, ID1 regulated
170	MEN2	0.83	205600_x_a	homeo box B5
171	MEN2	0.83	206833_s_a	acylphosphatase 2, muscle type
172	MEN2	0.83	204685_s_a	ATPase, Ca++ transporting, plasma membrane 2
173	MEN2	0.83	211691_x_a	accession number AF293339.1
174	MEN2	0.83	220405_at	syntrophin, gamma 1
175	MEN2	0.83	213695_at	paraoxonase 3
176	MEN2	0.83	204973_at	gap junction protein, beta 1, 32kDa (connexin 32
177	MEN2	0.83	43511_s_at	Homo sapiens mRNA; cDNA DKFZp762M127 (from clone DKFZp762M127
178	MEN2	0.83	219263_at	hypothetical protein FLJ23516
179	MEN2	0.83	201989_s_a	cAMP responsive element binding protein-like 2
180	MEN2	0.82	208448_x_a	interferon, alpha 16
181	MEN2	0.82	206506_s_a	suppressor of Ty 3 homolog (S. cerevisiae)
182	MEN2	0.82	33197_at	myosin VIIA (Usher syndrome 1B (autosomal recessive, severe)
183	MEN2	0.82	216366_x_a	accession number AF047245.1
184	MEN2	0.82	214140_at	hypothetical protein MGC39851
185	MEN2	0.82	47571_at	zinc finger protein 236
186	MEN2	0.82	209933_s_a	leukocyte membrane antigen
187	MEN2	0.82	208844_at	voltage-dependent anion channel 3
188	MEN2	0.81	206298_at	hypothetical protein from clones 23549 and 23762
189	MEN2	0.81	210686_x_a	solute carrier family 25 (mitochondrial carrier), member 16
190	MEN2	0.81	213343_s_a	hypothetical protein PP1665
191	MEN2	0.81	213992_at	collagen, type IV, alpha 6
192	MEN2	0.81	209057_x_a	CDC5 cell division cycle 5-like (S. pombe)
193	MEN2	0.81	203159_at	glutaminase
194	MEN2	0.81	221324_at	taste receptor, type 2, member 1
195	MEN2	0.81	210739_x_a	solute carrier family 4, sodium bicarbonate cotransporter, member 4
196	MEN2	0.81	203077_s_a	MAD, mothers against decapentaplegic homolog 2 (Drosophila
197	MEN2	0.81	209500_x_a	tumor necrosis factor (ligand) superfamily, member 13
198	MEN2	0.81	203328_x_a	insulin-degrading enzyme
199	MEN2	0.81	214180_at	Homo sapiens mRNA; cDNA DKFZp564H203 (from clone DKFZp564H203
200	MEN2	0.8	219255_x_a	interleukin 17B receptor
201	SDH	2.57	204597_x_a	stanniocalcin 1
202	SDH	1.96	209392_at	ectonucleotide pyrophosphatase/phosphodiesterase 2 (autotaxin
203	SDH	1.73	201867_s_a	transducin (beta)-like 1X-linked
204	SDH	1.68	218546_at	hypothetical protein FLJ14146
205	SDH	1.47	214096_s_a	serine hydroxymethyltransferase 2 (mitochondrial

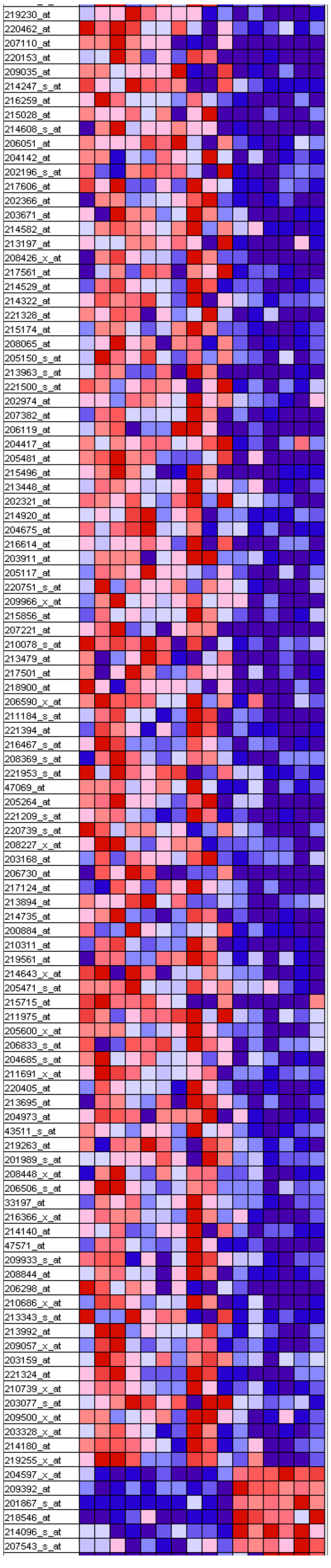

206	SDH	1.32	207543_s_a	procollagen-proline, 2-oxoglutarate 4-dioxygenase alpha polypeptide
207	SDH	1.31	204338_s_a	regulator of G-protein signalling 4
208	SDH	1.29	221933_at	neuroligin
209	SDH	1.28	213400_s_a	transducin (beta)-like 1X-linked
210	SDH	1.25	212037_at	pinin, desmosome associated protein
211	SDH	1.23	217673_x_a	accession number AA650558
212	SDH	1.22	221261_x_a	MAGE-E1 protein
213	SDH	1.21	202934_at	hexokinase 2
214	SDH	1.21	200094_s_a	eukaryotic translation elongation factor 2
215	SDH	1.21	201868_s_a	transducin (beta)-like 1X-linked
216	SDH	1.2	204337_at	regulator of G-protein signalling 4
217	SDH	1.19	217868_s_a	CGI-81 protein
218	SDH	1.18	201497_x_a	myosin, heavy polypeptide 11, smooth muscle
219	SDH	1.17	212997_s_a	tousled-like kinase 2
220	SDH	1.16	202499_s_a	solute carrier family 2 (facilitated glucose transporter), member 3
221	SDH	1.16	208932_at	protein phosphatase 4 (formerly X), catalytic subuni
222	SDH	1.14	201594_s_a	protein phosphatase 4, regulatory subunit 1
223	SDH	1.14	200941_at	heat shock factor binding protein 1
224	SDH	1.13	207961_x_a	myosin, heavy polypeptide 11, smooth muscle
225	SDH	1.13	219602_s_a	hypothetical protein FLJ23403
226	SDH	1.12	218317_x_a	hypothetical protein MGC5178
227	SDH	1.09	212348_s_a	KIAA0601 protein
228	SDH	1.08	203945_at	arginase, type II
229	SDH	1.08	200729_s_a	ARP2 actin-related protein 2 homolog (yeast
230	SDH	1.08	219791_s_a	hypothetical protein FLJ11539
231	SDH	1.07	203438_at	stanniocalcin 2
232	SDH	1.07	205603_s_a	diaphanous homolog 2 (Drosophila
233	SDH	1.07	206163_at	mab-21-like 1 (C. elegans)
234	SDH	1.07	205809_s_a	Wiskott-Aldrich syndrome-like
235	SDH	1.07	200944_s_a	high-mobility group nucleosome binding domain 1
236	SDH	1.05	823_at	chemokine (C-X3-C motif) ligand 1
237	SDH	1.04	204366_s_a	general transcription factor IIIC, polypeptide 2, beta 110kDa
238	SDH	1.04	218484_at	NADH:ubiquinone oxidoreductase MLRQ subunit homolog
239	SDH	1.04	214722_at	ESTs, Weakly similar to hypothetical protein FLJ20378
240	SDH	1.03	204904_at	gap junction protein, alpha 4, 37kDa (connexin 37
241	SDH	1.02	202133_at	transcriptional co-activator with PDZ-binding motif (TAZ
242	SDH	1.02	213011_s_a	triosephosphate isomerase 1
243	SDH	1.01	200790_at	ornithine decarboxylase 1
244	SDH	1.01	40560_at	T-box 2
245	SDH	1.01	213355_at	alpha2,3-sialyltransferase
246	SDH	1	204742_s_a	androgen-induced prostate proliferative shutoff associated protei
247	SDH	1	201182_s_a	chromodomain helicase DNA binding protein 4
248	SDH	0.98	205902_at	potassium intermediate/small conductance calcium-activated channel, subfamily
249	SDH	0.97	200012_x_a	ribosomal protein L21
250	SDH	0.97	208797_s_a	golgin-67
251	SDH	0.96	214803_at	Homo sapiens mRNA; cDNA DKFZp564N1116 (from clone DKFZp564N1116
252	SDH	0.96	209669_s_a	PAI-1 mRNA-binding protein
253	SDH	0.96	219862_s_a	nuclear prelamin A recognition factor
254	SDH	0.96	200745_s_a	guanine nucleotide binding protein (G protein), beta polypeptide
255	SDH	0.96	203006_at	inositol polyphosphate-5-phosphatase, 40kDa
256	SDH	0.95	202332_at	casein kinase 1, epsilon
257	SDH	0.95	204396_s_a	G protein-coupled receptor kinase 5
258	SDH	0.95	219165_at	PDZ-LIM protein mystique
259	SDH	0.95	205031_at	ephrin-B3
260	SDH	0.94	201215_at	plastin 3 (T isoform)
261	SDH	0.94	212876_at	UDP-Gal:betaGlcNAc beta 1,4- galactosyltransferase, polypeptide 4
262	SDH	0.94	208923_at	cytoplasmic FMR1 interacting protein 1
263	SDH	0.94	202112_at	von Willebrand factor
264	SDH	0.94	215111_s_a	transforming growth factor beta-stimulated protein TSC-22
265	SDH	0.94	212285_s_a	agrin
266	SDH	0.94	200934_at	DEK oncogene (DNA binding)
267	SDH	0.93	208693_s_a	glycyl-tRNA synthetase
268	SDH	0.93	202548_s_a	Rho guanine nucleotide exchange factor (GEF) 7
269	SDH	0.93	212273_x_a	GNAS complex locus
270	SDH	0.93	211700_s_a	trophinin
271	SDH	0.93	219024_at	pleckstrin homology domain containing, family A member 1
272	SDH	0.92	204115_at	guanine nucleotide binding protein 11
273	SDH	0.91	210776_x_a	transcription factor 3 (E2A immunoglobulin enhancer binding factors E12/E47
274	SDH	0.91	221878_at	Homo sapiens cDNA FLJ35653 fis, clone SPLEN2013690
275	SDH	0.91	210512_s_a	vascular endothelial growth factor
276	SDH	0.9	203708_at	phosphodiesterase 4B, cAMP-specific (phosphodiesterase E4 dunce homolog
277	SDH	0.9	221923_s_a	nucleophosmin (nucleolar phosphoprotein B23, numatrin
278	SDH	0.9	201054_at	heterogeneous nuclear ribonucleoprotein A0
279	SDH	0.9	214743_at	cut-like 1, CCAAT displacement protein (Drosophila
280	SDH	0.9	211651_s_a	accession number M20206.1
281	SDH	0.89	204622_x_a	nuclear receptor subfamily 4, group A, member 2
282	SDH	0.89	209024_s_a	NS1-associated protein 1
283	SDH	0.89	202233_s_a	ubiquinol-cytochrome c reductase hinge protein
284	SDH	0.88	200888_s_a	ribosomal protein L23
285	SDH	0.88	221726_at	ribosomal protein L22
286	SDH	0.88	206385_s_a	ankyrin 3, node of Ranvier (ankyrin G
287	SDH	0.88	201917_s_a	hypothetical protein FLJ10618
288	SDH	0.88	218834_s_a	hypothetical protein FLJ20539
289	SDH	0.87	205407_at	reversion-inducing-cysteine-rich protein with kazal motifs
290	SDH	0.87	201970_s_a	nuclear autoantigenic sperm protein (histone-binding
291	SDH	0.87	203098_at	chromodomain protein, Y chromosome-like
292	SDH	0.86	209137_s_a	ubiquitin specific protease 10
293	SDH	0.86	217419_x_a	agrin
294	SDH	0.85	218378_s_a	hypothetical protein FLJ13902
295	SDH	0.85	209162_s_a	PRP4 pre-mRNA processing factor 4 homolog (yeast)
296	SDH	0.85	209087_x_a	melanoma cell adhesion molecule
297	SDH	0.85	214039_s_a	putative integral membrane transporter
298	SDH	0.85	200679_x_a	high-mobility group box 1
299	SDH	0.85	201296_s_a	SOCS box-containing WD protein SWiP-1
300	SDH	0.85	217057_s_a	accession number AF107846
301	SDH	0.85	221766_s_a	chromosome 6 open reading frame 37
302	SDH	0.85	206481_s_a	LIM domain binding 2
303	SDH	0.84	200981_x_a	GNAS complex locus
304	SDH	0.84	201887_at	interleukin 13 receptor, alpha 1
305	SDH	0.84	207030_s_a	cysteine and glycine-rich protein 2
306	SDH	0.84	212865_s_a	collagen, type XIV, alpha 1 (undulin
307	SDH	0.84	205304_s_a	potassium inwardly-rectifying channel, subfamily J, member 8
308	SDH	0.84	201761_at	methylene tetrahydrofolate dehydrogenase (NAD+ dependent
309	SDH	0.84	201505_at	laminin, beta 1
310	SDH	0.84	209820_s_a	transducin (beta)-like 3
311	SDH	0.83	202208_s_a	ADP-ribosylation factor-like 7
312	SDH	0.83	208728_s_a	cell division cycle 42 (GTP binding protein, 25kDa

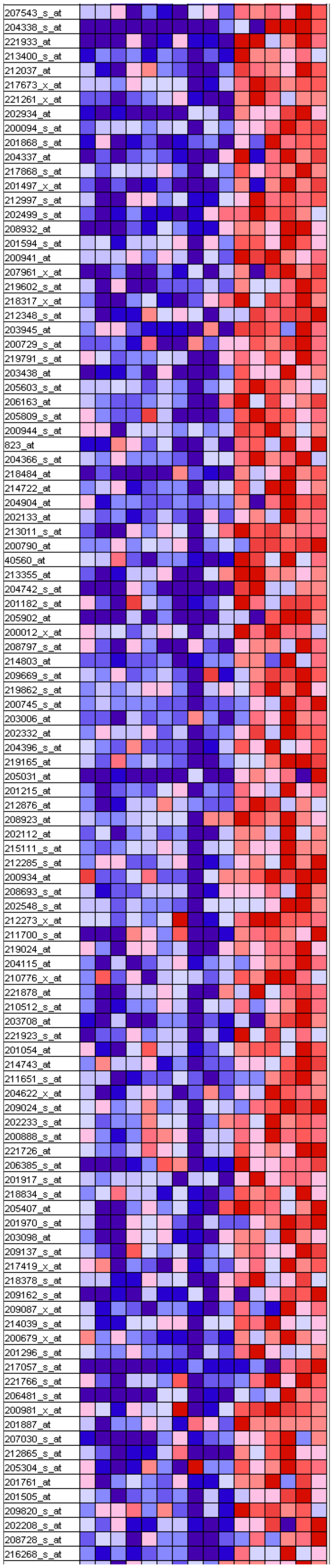

313	SDH	0.83	216268_s_a	jagged 1 (Alagille syndrome)
314	SDH	0.83	221502_at	karyopherin alpha 3 (importin alpha 4
315	SDH	0.82	210999_s_a	growth factor receptor-bound protein 10
316	SDH	0.82	221427_s_a	likely ortholog of mouse Paneth cell enhanced expressio
317	SDH	0.82	213401_s_a	transducin (beta)-like 1X-linked
318	SDH	0.82	216331_at	integrin, alpha 7
319	SDH	0.82	213230_at	paraneoplastic antigen
320	SDH	0.82	203946_s_a	arginase, type II
321	SDH	0.82	208981_at	platelet/endothelial cell adhesion molecule (CD31 antigen
322	SDH	0.82	205224_at	surfeit 2
323	SDH	0.82	204472_at	GTP binding protein overexpressed in skeletal muscle
324	SDH	0.81	218258_at	RNA polymerase I 16 kDa subuni
325	SDH	0.81	209210_s_a	mitogen inducible 2
326	SDH	0.81	208798_x_a	golgin-67
327	SDH	0.81	209161_at	PRP4 pre-mRNA processing factor 4 homolog (yeast)
328	SDH	0.81	200878_at	Homo sapiens clone 23698 mRNA sequence
329	SDH	0.81	207966_s_a	golgi apparatus protein 1
330	SDH	0.81	52255_s_at	collagen, type V, alpha 3
331	SDH	0.81	213301_x_a	transcriptional intermediary factor 1
332	SDH	0.8	213268_at	accession number Z98884
333	SDH	0.8	208803_s_a	signal recognition particle 72kDa
334	SDH	0.8	206397_x_a	growth differentiation factor 1
335	SDH	0.8	218137_s_a	stromal membrane-associated protein
336	SDH	0.8	202146_at	interferon-related developmental regulator 1
337	SDH	0.8	211503_s_a	RAB14, member RAS oncogene family
338	SDH	0.8	213491_x_a	ribophorin II
339	SDH	0.8	213940_s_a	formin-binding protein 17
340	SDH	0.8	209655_s_a	brain cell membrane protein 1
341	SDH	0.8	205463_s_a	platelet-derived growth factor alpha polypeptide
342	SDH	0.8	201209_at	histone deacetylase 1
343	SDH	0.8	201551_s_a	lysosomal-associated membrane protein 1
344	SDH	0.8	201216_at	chromosome 12 open reading frame 8
345	SDH	0.8	221830_at	Homo sapiens cDNA FLJ37267 fis, clone BRAMY201129
346	SDH	0.79	201540_at	four and a half LIM domains 1
347	SDH	0.79	219532_at	elongation of very long chain fatty acids (FEN1/Elo2, SUR4/Elo3, yeast)-like
348	SDH	0.79	202672_s_a	activating transcription factor 3
349	SDH	0.79	218974_at	hypothetical protein FLJ10159
350	SDH	0.79	204078_at	nucleolar autoantigen (55kD) similar to rat synaptonemal complex protei
351	SDH	0.79	202458_at	protease, serine, 23
352	SDH	0.79	200746_s_a	guanine nucleotide binding protein (G protein), beta polypeptide
353	SDH	0.78	200598_s_a	tumor rejection antigen (gp96) 1
354	SDH	0.78	200606_at	desmoplakin (DPI, DPII)
355	SDH	0.78	204937_s_a	zinc finger protein 274
356	SDH	0.78	201496_x_a	myosin, heavy polypeptide 11, smooth muscle
357	SDH	0.78	205656_at	protocadherin 17
358	SDH	0.78	219249_s_a	FK506 binding protein 10, 65 kDa
359	SDH	0.77	208661_s_a	tetratricopeptide repeat domain 3
360	SDH	0.77	220584_at	hypothetical protein FLJ22184
361	SDH	0.77	221560_at	MAP/microtubule affinity-regulating kinase 4
362	SDH	0.77	40472_at	Homo sapiens clone 23763 unknown mRNA, partial cds
363	SDH	0.77	209337_at	PC4 and SFRS1 interacting protein 2
364	SDH	0.77	208775_at	exportin 1 (CRM1 homolog, yeast)
365	SDH	0.77	210095_s_a	insulin-like growth factor binding protein 3
366	SDH	0.77	201190_s_a	phosphotidylinositol transfer protein
367	SDH	0.77	218669_at	hypothetical protein similar to small G proteins, especially RAP-2A
368	SDH	0.77	204339_s_a	regulator of G-protein signalling 4
369	SDH	0.76	210407_at	protein phosphatase 1A (formerly 2C), magnesium-dependent, alpha isoform
370	SDH	0.76	200999_s_a	cytoskeleton-associated protein 4
371	SDH	0.76	209281_s_a	ATPase, Ca++ transporting, plasma membrane 1
372	SDH	0.76	220694_at	HSPC054 protein
373	SDH	0.76	212979_s_a	KIAA0738 gene product
374	SDH	0.76	212967_x_a	nucleosome assembly protein 1-like 1
375	SDH	0.76	212730_at	desmuslin
376	SDH	0.75	212643_at	likely ortholog of mouse MAPK-interacting and spindle-stabilizing protei
377	SDH	0.75	209099_x_a	jagged 1 (Alagille syndrome)
378	SDH	0.75	211564_s_a	LIM domain protein
379	SDH	0.75	213901_x_a	RNA binding motif protein 9
380	SDH	0.75	200632_s_a	N-myc downstream regulated gene 1
381	SDH	0.75	201125_s_a	integrin, beta 5
382	SDH	0.75	200755_s_a	calumenin
383	SDH	0.74	204099_at	SWI/SNF related, matrix associated,regulator of chromatin, subfamily d, member 3
384	SDH	0.74	204464_s_a	endothelin receptor type A
385	SDH	0.74	208985_s_a	eukaryotic translation initiation factor 3, subunit 1 alpha, 35kD
386	SDH	0.74	203749_s_a	retinoic acid receptor, alpha
387	SDH	0.74	204621_s_a	nuclear receptor subfamily 4, group A, member 2
388	SDH	0.74	205303_at	potassium inwardly-rectifying channel, subfamily J, member 8
389	SDH	0.74	200713_s_a	microtubule-associated protein, RP/EB family, member 1
390	SDH	0.74	218181_s_a	hypothetical protein FLJ20373
391	SDH	0.74	214093_s_a	Homo sapiens mRNA; cDNA DKFZp564O0122 (from clone DKFZp564O0122
392	SDH	0.74	201892_s_a	IMP (inosine monophosphate) dehydrogenase 2
393	SDH	0.74	221846_s_a	cask-interacting protein 2
394	SDH	0.73	208900_s_a	topoisomerase (DNA) I
395	SDH	0.73	200650_s_a	lactate dehydrogenase A
396	SDH	0.73	203238_s_a	Notch homolog 3 (Drosophila)
397	SDH	0.73	205572_at	angiopoietin 2
398	SDH	0.73	202082_s_a	SEC14-like 1 (S. cerevisiae)
399	SDH	0.73	212759_s_a	transcription factor 7-like 2 (T-cell specific, HMG-box
400	SDH	0.73	211542_x_a	ribosomal protein S10

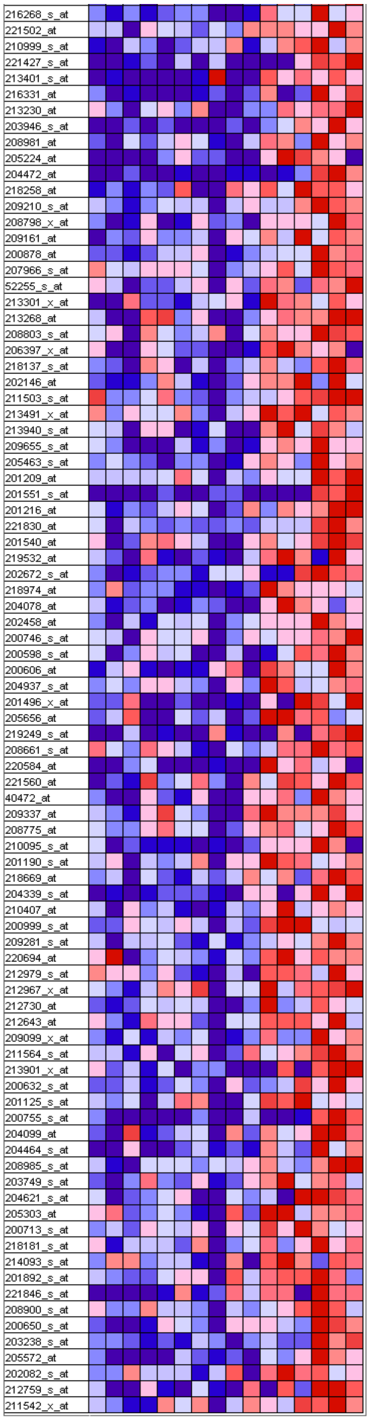

Application of the two-class prediction models to a test set made up of the entire dataset

MEN2 vs. VHL model		MEN2 vs. SDH model		FPT vs. VHL model		FPT vs. SDH model			
Sample	Predicted	Sample	Predicted	Sample	Predicted	Sample	Predicted	Observed	Cluster mem
P101	VHL	P101	SDH	P101	VHL	P101	SDH	B_SDHB	1
P103	MEN2	P103	MEN2	P103	FPT	P103	FPT	MEN2A	2
P105	MEN2	P105	MEN2	P105	FPT	P105	FPT	SPOR	2
P106	MEN2	P106	MEN2	P106	FPT	P106	FPT	MEN2A	2
P107	VHL	P107	SDH	P107	VHL	P107	SDH	B_SDHB	1
P108	MEN2	P108	MEN2	P108	FPT	P108	FPT	SPOR	2
P111	MEN2	P111	MEN2	P111	FPT	P111	FPT	SPOR	2
P112	VHL	P112	MEN2	P112	FPT	P112	FPT	SPOR	2
P115	MEN2	P115	MEN2	P115	FPT	P115	FPT	MEN2A	2
P120	VHL	P120	SDH	P120	VHL	P120	SDH	SPOR	1
P122	MEN2	P122	MEN2	P122	FPT	P122	FPT	SPOR	2
P128	VHL	P128	MEN2	P128	VHL	P128	SDH	SPOR	1
P136	VHL	P136	SDH	P136	VHL	P136	SDH	B_SDHB	1
P140	VHL	P140	SDH	P140	VHL	P140	SDH	D_SDHD	1
P142	VHL	P142	SDH	P142	VHL	P142	SDH	VHL	1
P143	MEN2	P143	MEN2	P143	FPT	P143	FPT	NF1	2
P144	VHL	P144	SDH	P144	VHL	P144	FPT	SPOR	2
P145	MEN2	P145	MEN2	P145	FPT	P145	FPT	FP2	2
P146	MEN2	P146	MEN2	P146	FPT	P146	FPT	SPOR	2
P148	MEN2	P148	MEN2	P148	FPT	P148	FPT	SPOR	2
P149	MEN2	P149	MEN2	P149	FPT	P149	FPT	FP2	2
P150	MEN2	P150	MEN2	P150	FPT	P150	FPT	SPOR	2
P152	VHL	P152	SDH	P152	VHL	P152	SDH	FP3	1
P153	VHL	P153	SDH	P153	VHL	P153	SDH	VHL	1
P154	VHL	P154	SDH	P154	VHL	P154	SDH	VHL	1
P155	VHL	P155	MEN2	P155	VHL	P155	SDH	VHL	1
P156	VHL	P156	SDH	P156	VHL	P156	SDH	VHL	1
P157	MEN2	P157	MEN2	P157	VHL	P157	FPT	SPOR	2
P158	VHL	P158	SDH	P158	VHL	P158	SDH	SPOR	1
P16	VHL	P16	SDH	P16	VHL	P16	SDH	SPOR	1
P166	MEN2	P166	MEN2	P166	FPT	P166	FPT	SPOR	2
P167	MEN2	P167	MEN2	P167	FPT	P167	FPT	NF1	2
P171	MEN2	P171	MEN2	P171	FPT	P171	FPT	FP1	2
P172	MEN2	P172	MEN2	P172	FPT	P172	FPT	SPOR	2
P173	MEN2	P173	MEN2	P173	FPT	P173	FPT	SPOR	2
P174	MEN2	P174	MEN2	P174	FPT	P174	FPT	SPOR	2
P175	MEN2	P175	MEN2	P175	VHL	P175	FPT	SPOR	2
P176	MEN2	P176	MEN2	P176	FPT	P176	FPT	SPOR	2
P177	MEN2	P177	MEN2	P177	FPT	P177	FPT	SPOR	2
P178	MEN2	P178	MEN2	P178	FPT	P178	FPT	SPOR	2
P181	MEN2	P181	MEN2	P181	FPT	P181	FPT	SPOR	2
P182	MEN2	P182	MEN2	P182	FPT	P182	FPT	MEN2A	2
P188	MEN2	P188	MEN2	P188	FPT	P188	FPT	SPOR	2
P192	VHL	P192	MEN2	P192	VHL	P192	SDH	VHL	1
P195	MEN2	P195	MEN2	P195	FPT	P195	FPT	FP2	2
P196	MEN2	P196	MEN2	P196	FPT	P196	FPT	FP4	2
P197	MEN2	P197	MEN2	P197	FPT	P197	FPT	FP4	2
P198	MEN2	P198	MEN2	P198	FPT	P198	FPT	MEN2A	2
P203	VHL	P203	SDH	P203	VHL	P203	FPT	SPOR	1
P204	MEN2	P204	MEN2	P204	FPT	P204	FPT	SPOR	2
P205	MEN2	P205	MEN2	P205	FPT	P205	FPT	MEN2A	2
P207	MEN2	P207	MEN2	P207	FPT	P207	FPT	MEN2A	2
P208	VHL	P208	SDH	P208	VHL	P208	SDH	SPOR	2
P209	VHL	P209	SDH	P209	VHL	P209	SDH	SPOR	2
P210	MEN2	P210	MEN2	P210	FPT	P210	FPT	MEN2A	2
P215	MEN2	P215	MEN2	P215	FPT	P215	FPT	FP5	2
P216	MEN2	P216	SDH	P216	FPT	P216	SDH	SPOR	1
P218	MEN2	P218	MEN2	P218	FPT	P218	FPT	SPOR	2
P219	VHL	P219	SDH	P219	VHL	P219	SDH	SPOR	1
P220	VHL	P220	SDH	P220	VHL	P220	SDH	FP7	1
P26	MEN2	P26	MEN2	P26	FPT	P26	FPT	FP1	2
P27	MEN2	P27	MEN2	P27	FPT	P27	FPT	FP1	2
P29	VHL	P29	MEN2	P29	FPT	P29	FPT	FP1	2
P37	VHL	P37	SDH	P37	VHL	P37	SDH	SPOR	1
P54	MEN2	P54	MEN2	P54	FPT	P54	FPT	SPOR	2
P55	MEN2	P55	MEN2	P55	FPT	P55	FPT	SPOR	2
P56	MEN2	P56	MEN2	P56	FPT	P56	FPT	SPOR	2
P58	VHL	P58	SDH	P58	VHL	P58	SDH	B_SDHB	1
P61	MEN2	P61	MEN2	P61	FPT	P61	FPT	MEN2A	2
P76	VHL	P76	MEN2	P76	FPT	P76	FPT	FP6	2
P85	VHL	P85	SDH	P85	VHL	P85	SDH	SPOR	1
P87	MEN2	P87	MEN2	P87	FPT	P87	FPT	SPOR	2
P89	VHL	P89	SDH	P89	VHL	P89	SDH	B_SDHB	1
P91	MEN2	P91	MEN2	P91	FPT	P91	FPT	MEN2A	2
P92	VHL	P92	SDH	P92	VHL	P92	SDH	FP3	1
P93	VHL	P93	SDH	P93	VHL	P93	SDH	FP3	1
% correct p	92%		93%		93%		95%		

Yellow highlight=incorrect predictions
