# Supplementary material for: A HIF1α Regulatory Loop Links Hypoxia and Mitochondrial Signals in Pheochromocytomas
Source: PLoS Genet. 2005 Jul 25;1(1):e8. doi: 10.1371/journal.pgen.0010008 (PMC1183527; doi:10.1371/journal.pgen.0010008)
Supplement: Dataset S5 — (12 KB PDF) [file pgen.0010008.sd005.pdf]

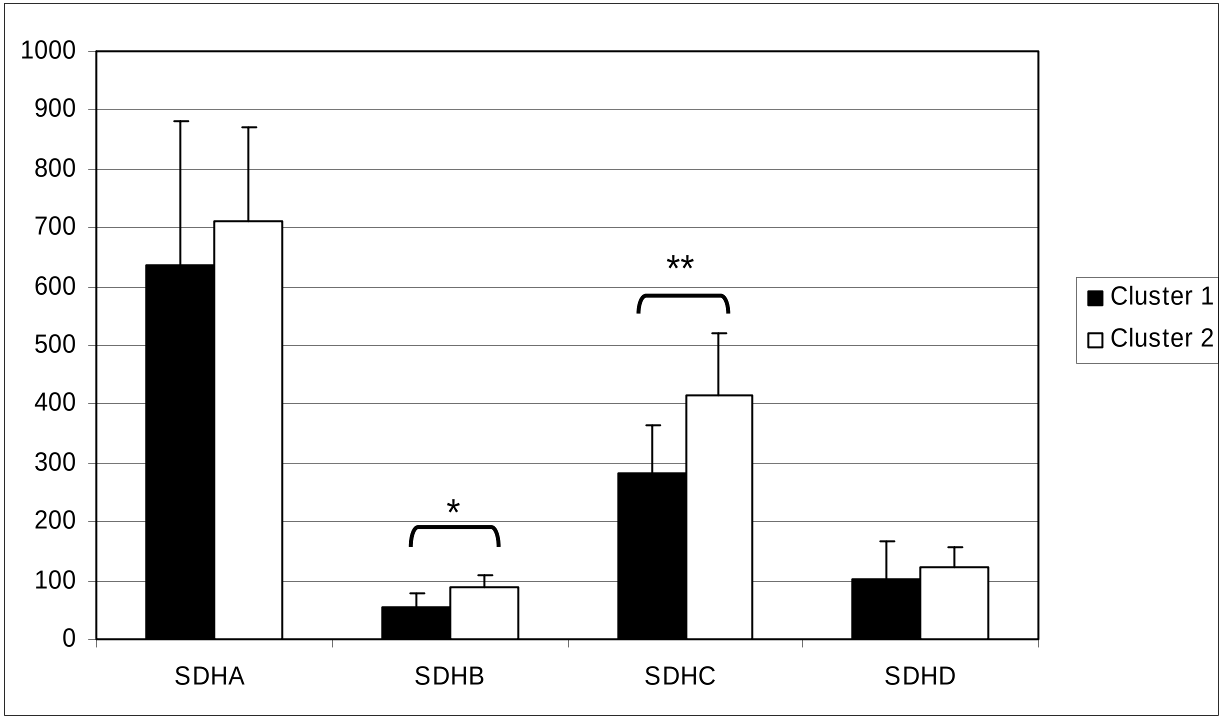

Supplementary Data 5. mRNA expression of the four subunits of complex II by U133 array in pheochromocytomas from cluster 1 and cluster 2. Results shown are the average of signal from multiple probes for each gene after normalization (see Methods for Details). * p<0.001; ** p<0.01
